# Identification of *Uncaria rhynchophylla* in the Potential Treatment of Alzheimer’s Disease by Integrating Virtual Screening and In Vitro Validation

**DOI:** 10.3390/ijms242015457

**Published:** 2023-10-22

**Authors:** Shuang Jiang, Gilwa Borjigin, Jiahui Sun, Qi Li, Qianbo Wang, Yuanqiu Mu, Xuepeng Shi, Qian Li, Xiaotong Wang, Xiaodan Song, Zhibin Wang, Chunjuan Yang

**Affiliations:** 1 Department of Pharmaceutical Analysis and Analytical Chemistry, College of Pharmacy, Harbin Medical University, Harbin 150081, China; jiangshuang@hrbmu.edu.cn (S.J.); borjigingilwa@hrbmu.edu.cn (G.B.); 202101089@hrbmu.edu.cn (J.S.); meshilq@outlook.com (Q.L.); wqb-1230@163.com (Q.W.); myqj135790@163.com (Y.M.); sxp08031026@163.com (X.S.); liqian@ems.hrbmu.edu.cn (Q.L.); wangxt61@163.com (X.W.); danxsong@163.com (X.S.); 2 Key Laboratory of Chinese Materia Medica, Ministry of Education, Heilongjiang University of Chinese Medicine, Harbin 150040, China; wzbmailbox@126.com; 3 Key Laboratory of Gut Microbiota and Pharmacogenomics of Heilongjiang Province, College of Pharmacy, Harbin Medical University, Harbin 150081, China

**Keywords:** Alzheimer’s disease, *Uncaria rhynchophylla*, network pharmacology, p-tau

## Abstract

*Uncaria rhynchophylla* (Gouteng in Chinese, GT) is the main medicine in many traditional recipes in China. It is commonly used to alleviate central nervous system (CNS) disorders, although its mechanism in Alzheimer’s disease is still unknown. This study was designed to predict and validate the underlying mechanism in AD treatment, thus illustrating the biological mechanisms of GT in treating AD. In this study, a PPI network was constructed, KEGG analysis and GO analysis were performed, and an “active ingredient-target-pathway” network for the treatment of Alzheimer’s disease was constructed. The active ingredients of GT were screened out, and the key targets were performed by molecular docking. UHPLC-Q-Exactive Orbitrap MS was used to screen the main active ingredients and was compared with the network pharmacology results, which verified that GT did contain the above ingredients. A total of targets were found to be significantly bound up with tau, Aβ, or Aβ and tau through the network pharmacology study. Three SH-SY5Y cell models induced by okadaic acid (OA), Na_2_S_2_O_4_, and H_2_O_2_ were established for in vitro validation. We first found that GT can reverse the increase in the hyperphosphorylation of tau induced by OA to some extent, protecting against ROS damage. Moreover, the results also indicated that GT has significant neuroprotective effects. This study provides a basis for studying the potential mechanisms of GT in the treatment of AD.

## 1. Introduction

Alzheimer’s disease (AD), known as “dementia” in ancient China, first appeared in the medical book “Hua Tuo Shen Yi Mi Zhuan” (Han Dynasty) and is a neurodegenerative disease whose pathogenesis is genetically related to multifactorial induction [1,2]. The distinguishing feature of AD patients is the presence of beta-amyloid and tau proteins, which accumulate in the brain and hinder normal cognitive functions. The clinical manifestations include memory impairment, apraxia, agnosia, aphasia, executive dysfunction, impairment of visuospatial skills, and personality and behavioral changes [3,4]. According to the statistics of the World Alzheimer Report 2021, dementia is now the seventh leading cause of mortality globally, and AD is associated with the highest cost to society. A recent report indicated that the number of patients with dementia will triple worldwide by 2050 (about 153 million). It has become a top priority to develop effective methods and drugs for the treatment of AD and improvement of the pathological process of AD patients. At present, the pathogenesis of AD is still not clear; large numbers of clinical reports show that Aβ protein deposition, abnormally hyperphosphorylated tau protein, oxidative stress, neuroinflammation, and apoptosis contribute to its pathogenesis [5,6,7,8]. Moreover, research has targeted systemic innate immune cells, which may be a therapeutic avenue for AD [9]. So far, more than 200 clinical trials of drugs for AD treatment have been stopped due to efficacy problems [10], and currently, there is no effective treatment [8]. The pathogenesis of AD is complex, which creates considerable difficulties in the development of an effective drug. The drugs for treating AD approved by the FDA include donepezil, rivastigmine, galanthamine base, and memantine hydrochloride [11]. However, these drugs can only temporarily improve the cognitive impairment, and they can not prevent or delay the pathological process of AD. They also have side effects, including nausea and vomiting. The FDA has more recently approved aducanumab, an antibody that targets Aβ, which was used to treat AD (June 2021); however, there is great controversy regarding the curative effect [12,13]. Therefore, finding effective drugs to prevent or treat AD remains an urgent problem.

The earliest records of the medicinal use of *Uncaria rhynchophylla* (Mig.) Miq. ex Havil (*Uncaria rhynchophylla*, GT) were during the Ming Dynasty, and they are now included in the first part of the Chinese Pharmacopoeia. GT was traditionally often used to treat CNS disorders, including convulsions and epilepsy (China Pharmacopoeia Committee, 2020). GT is the main ingredient in many classic formulas for treating CNS disorders, such as Gou-teng-san, Tian-ma-gou-teng-yin, and Ling-jiao-gou-teng-tang [14,15,16]. GT alkaloids are its main active ingredients, which have remarkable efficacy in the treatment of CNS diseases. They have been proven to have neuroprotective effects against Parkinson’s disease and AD through a variety of mechanisms, including antioxidant effects, anti-inflammatory effects, neurotransmitter regulation, and intracellular calcium overloading [17,18].

In this study, various databases, such as TCMSP, TCMID, TCMIP, and BATMAN-TCM, were used to predict the relevant targets of AD and GT, explore the potential active ingredients for the prevention and treatment of AD, and validate the findings with cellular modeling in vitro. The pharmacological mechanisms associated with GT and AD were predicted using network pharmacology techniques and validated in vitro for the first time to provide a methodological basis for network pharmacology to predict the pharmacological mechanisms of AD and experimentally carry out validation, as well providing a theoretical basis for the prevention and treatment of AD. Can GT be the potential drug in future AD research?

## 2. Results

### 2.1. Network Pharmacology Analysis

#### 2.1.1. Active Compounds

The databases (TCMSP, TCMID, TCMIP, and BATMAN-TCM) provided a total of 246 chemical components for GT. According to the SwissADME system (Table S2), 42 alkaloids were obtained, as shown in Table S1.

#### 2.1.2. Compounds and Target Screening

**Figure 1:**
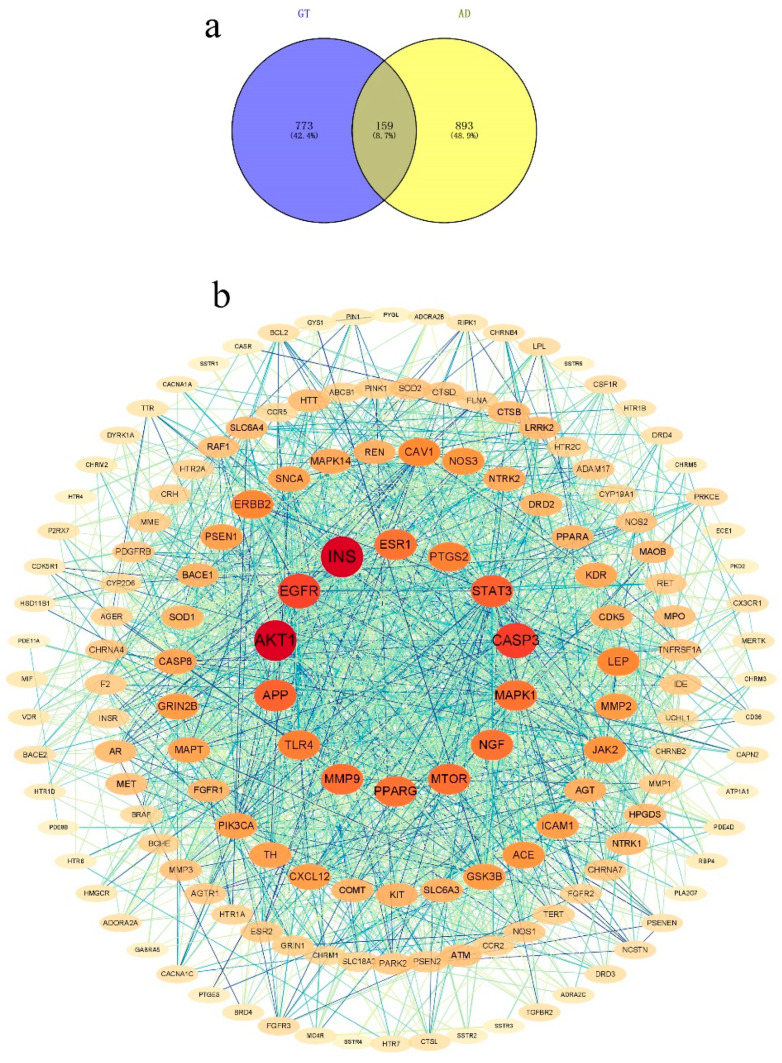
Network analysis of the related targets of GT and AD. (**a**) Venn diagram of ingredient targets of GT- and AD-related targets. (**b**) The PPI network of the related targets of GT and AD. The size of circles represented the degree of value.

The targets were obtained according to the respective target predictions from the databases (SEA, Swiss Target Prediction, TCMSP, and BATMAN-TCM) for GT and the databases (DisGeNET, GeneCards, OMIM, and TTD) for AD. A total of 159 relevant targets of GT and AD were obtained, and the details can be found in Table S3 and Figure 1a.

#### 2.1.3. PPI Network

The PPI network for potential targets (GT) in AD treatment was obtained from the STRING database. It consisted of 159 nodes and 1911 edges (Figure 1b). Nodes with a degree of >48.40, twice the average number of neighbors, were identified as key nodes in the network. (Table 1). The results showed that there were 17 key targets in the PPI network, and INS and AKT1 serine had the highest degree values (>90). This suggested that GT can significantly affect AD via the above 17 key targets.

#### 2.1.4. Construction of a Potential Ingredient Target-AD Target-Pathway Network

**Figure 2:**
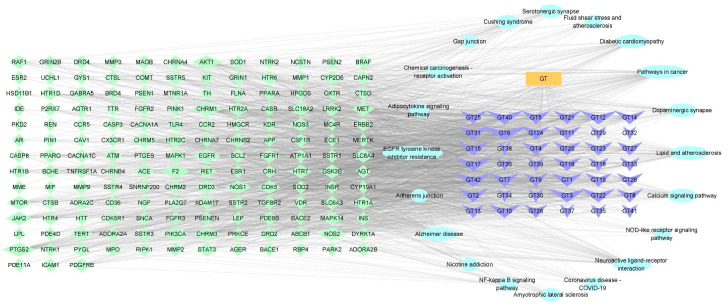
The “ingredient-targets-pathways” network. The blue V shapes represent the ingredients of GT. The green diamonds represent related targets of GT and AD, and the light blue ellipses represent pathways. GT: *Uncaria rhynchophylla*. The number of GT represents the ingredients of GT, and the names of the GT ingredients are shown in Table S1.

The network of GT against AD is depicted in Figure 2. Based on the above results, a potential component target-AD target-pathway network with 222 nodes and 1639 edges was formed using 159 targets, 42 alkaloids, and 20 signaling pathways, with the aim of revealing the therapeutic mechanism of GT ingredients in AD. The active components corresponded to multiple targets, which indicated that the mechanism of the treatment of AD by GT is a multi-ingredient and multi-target treatment.

#### 2.1.5. Enrichment Analysis

**Figure 3:**
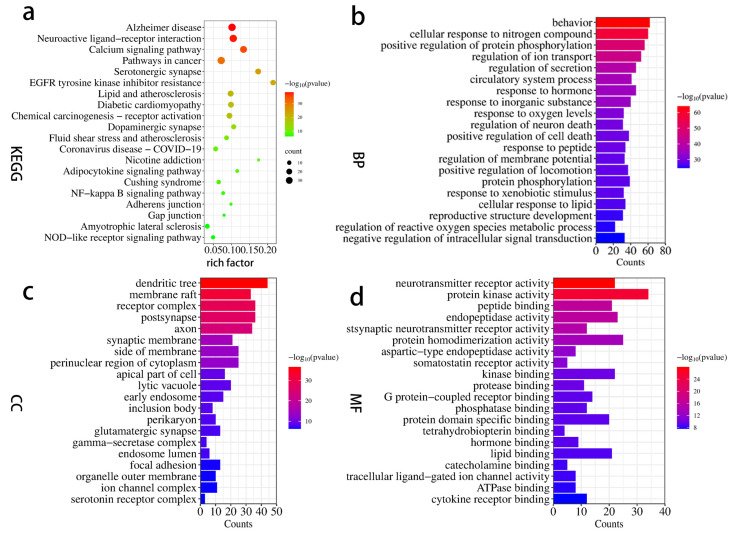
The enrichment analysis of GO and KEGG of GT and AD. (**a**) KEGG pathway, (**b**) biological process (BP), (**c**) cellular component (CC), and (**d**) molecular function (MF).

To identify signaling pathways, the KEGG enrichment analysis was obtained with the 159 targets and “human sapiens” in the metascape system. The KEGG pathways mainly involved Alzheimer’s disease (hsa05010), neuroactive ligand-receptor interaction (hsa04080), calcium signaling pathway (sa04020), pathways in cancer (hsa05200), serotonergic synapse (hsa04726), and EGFR tyrosine kinase inhibitor resistance (hsa01521) (Figure 3a, Table S4).

The enrichment results of the pathways showed that the Alzheimer disease pathway (hsa05010, *p* = 9.16 × 10^−39^, degree = 39) was the most effective. The GO enrichment analysis included biological processes (BPs), molecular functions (MFs), and cellular components (CCs), which related to the cellular response to nitrogen compounds, behavior, positive regulation of protein phosphorylation, and other biological processes. The results are presented in Figure 3. All the enrichment results indicated that GT has potential efficacy for the treatment of AD via different pathways.

#### 2.1.6. Screening of Bioactive Compounds by UHPLC-Q-Exactive Orbitrap MS Analysis 

**Figure 4:**
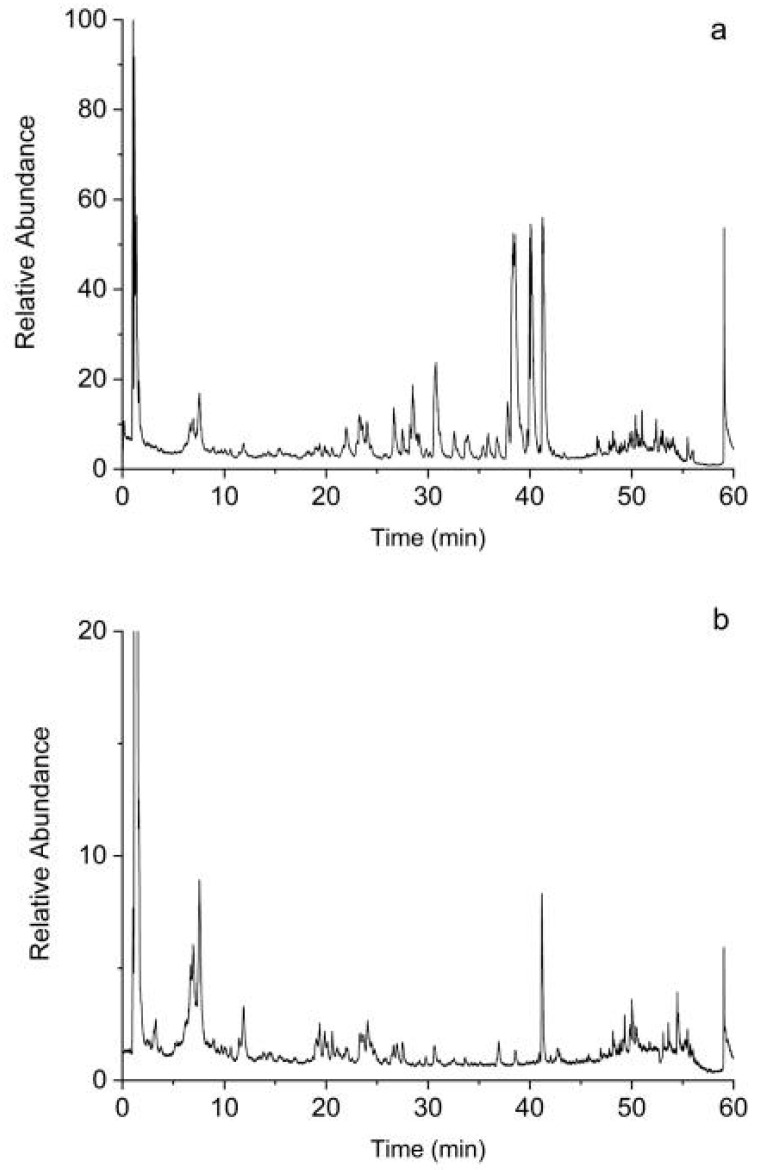
The UHPLC-Q-Exactive Orbitrap MS base peak intensity chromatograms of GT. (**a**) Positive mode. (**b**) Negative mode.

The compounds in GT were identified (Table 2). Figure 4 shows the base peak intensity chromatograms of GT in positive and negative modes. The present approach identified 80 bioactive compounds in GT, including 35 of 42 components based on the databases (Table 2 and Table S6). The “component target-AD target-pathway” network identified some important bioactive compounds, such as corynanthine, angustidine, yohimbine, tetrahydroalstonine, dihydrocorynantheine, hirsutine, hirsuteine, rhynchophylline, and isorhynchophylline. 

#### 2.1.7. Molecular Docking

**Figure 5:**
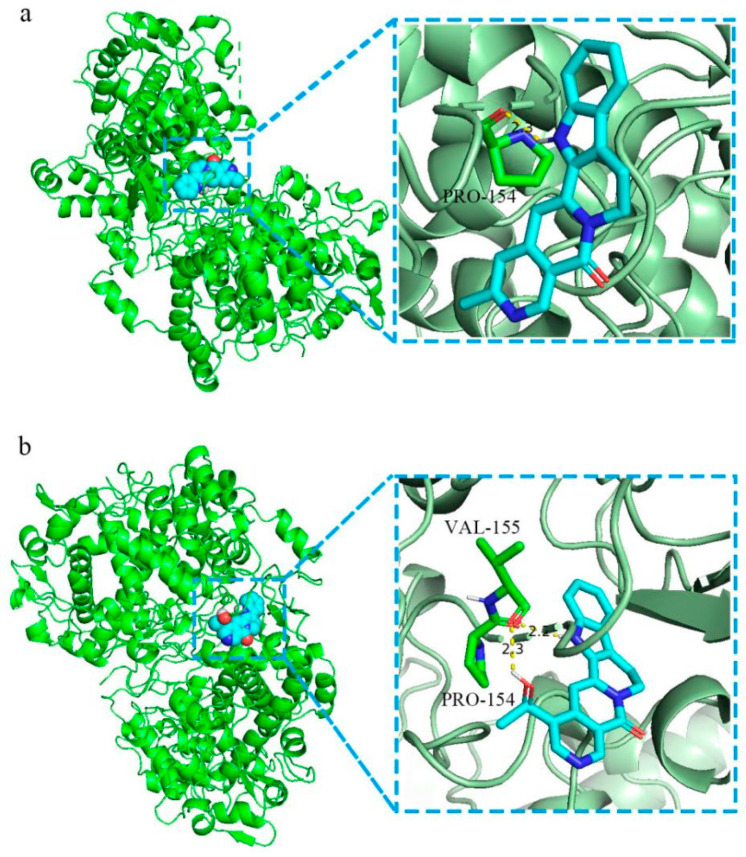
Schematic diagram of angustidine and angustoline docking with the core target. (**a**) PTGS2 docking with angustidine. (**b**) PTGS2 docking with angustoline.

Molecular docking was performed with GT ingredients and the key targets of the PPI network. The more stable the docking module, the lower the binding energy. The PPI network diagram revealed that GT can significantly affect AD through 17 key targets. Then, we carried out molecular docking between the 17 key targets and GT components. As shown in Figure 5, the molecular docking of PTGS2 with angustidine and angustoline had lower binding energy, and the docking modules were more stable. Angustoline interacts with PTGS2 through two amino acid residues, namely VAL-155 and PRO-154, with a binding energy of 9.7 kcal/mol < −5.0 kcal/mol. Angustidine interacted with PTGS2 through one amino acid residue, namely PRO-154, with a binding energy of 9.6 kcal/mol < −5.0 kcal/mol. The binding energy docking information is listed in Table S5.

#### 2.1.8. Bioinformatics Analysis of Targets of the Ingredients of GT Related to Aβ and Tau Pathology

**Figure 6:**
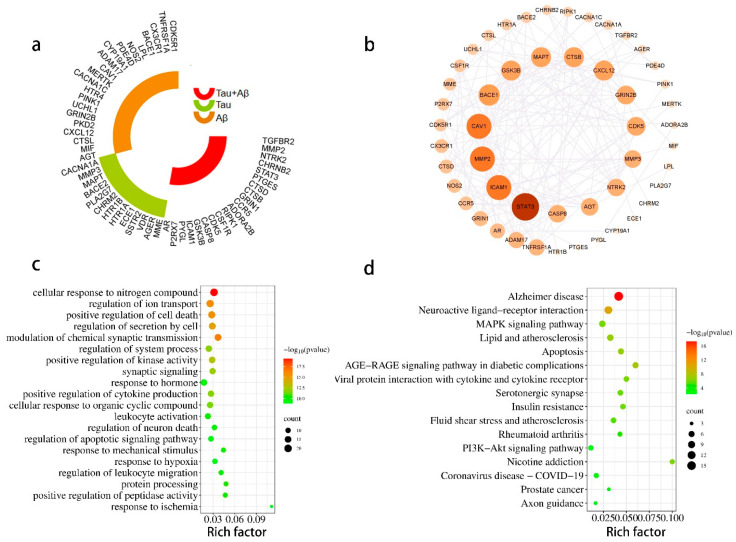
Bioinformatics analysis of GT targets related to tau, Aβ, or Aβ and tau pathology. (**a**) The targets of GT alkaloids are related to tau, Aβ, or Aβ and tau. (**b**) PPI network of proteins associated with the pathology of tau, Aβ, or Aβ and tau. (**c**) The top 20 GO BP enrichment analysis related to tau, Aβ, or Aβ and tau. (**d**) KEGG pathway enrichment analysis related to tau, Aβ, or Aβ and tau.

The AlzData database [19,20], which is a high-throughput data collection of AD, was utilized to investigate the relationship between potential targets of GT ingredients and Aβ and tau pathology. Figure 6a shows that out of 159 targets, 54 were significantly bound to tau, Aβ, or both. TNFRSF1A, CDK5R1, CX3CR1, BACE1, ADAM17, CAV1, and GRIN2B were significantly related to Aβ pathology. MMP3, CACNA1A, MAPT, BACE2, PLA2G7, and CHRM2 were significantly related to tau pathology. AR, GSK3B, CASP8, CDK5, CCR5, STAT3, TGFBR2, and MMP2 were significantly associated with Aβ and tau pathology. The PPI network constructs with 54 targets identified by the STRING database had 54 nodes and 190 edges (Figure 6b). STAT3, ICAM1, MMP2, CAV1, BACE1, GSK3B, MAPT, CTSB, CXCL12, GRIN2B, CDK5, MMP3, NTRK2, AGT, and CASP8 were considered as the key targets by degree.

**Figure 7:**
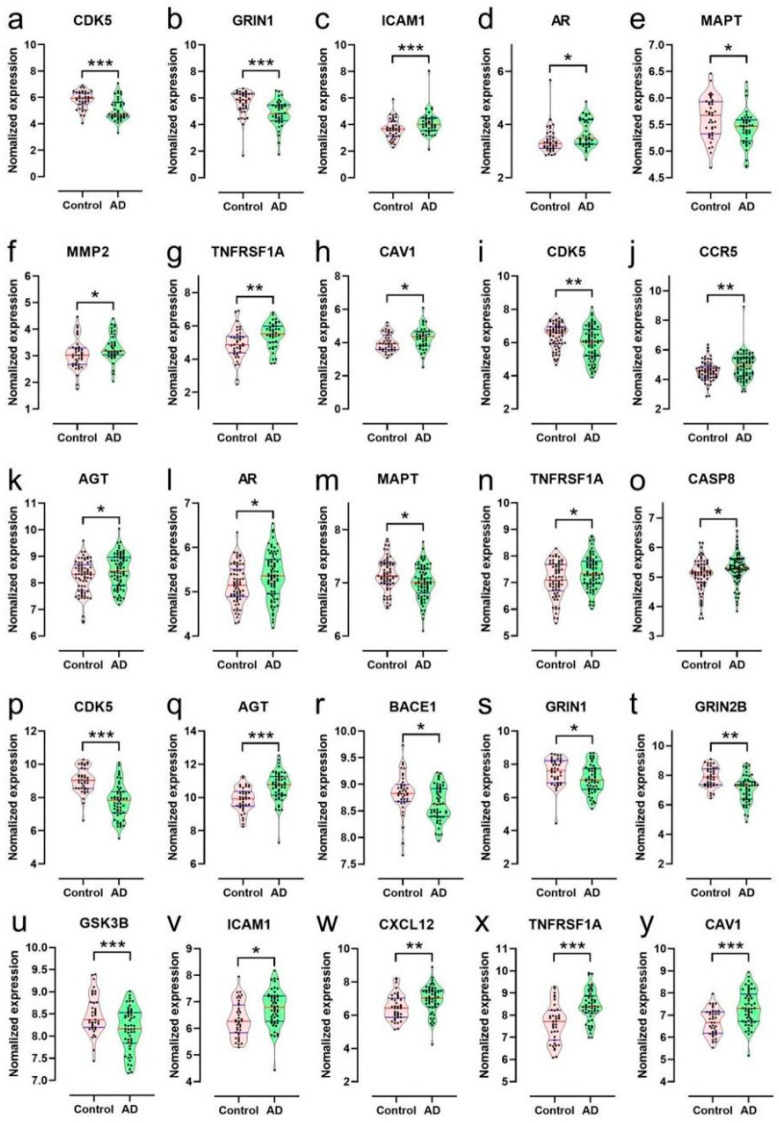
(**a**–**y**) The expression of the targets of GT related to tau, Aβ, or Aβ and tau pathology against AD in the entorhinal cortex or hippocampus or temporal cortex in the control and AD groups of the GEO dataset. Entorhinal cortex (**a**–**h**), *n* = 39 in each group. Hippocampus (**i**–**o**), *n* = 66 in the healthy control group and *n* = 74 in the AD group. Temporal cortex (**p**–**y**), *n* = 39 in the healthy control group and *n* = 52 in the AD group (* *p* < 0.05, ** *p* < 0.01, *** *p* < 0.001 compared with control group).

The GO BP and KEGG were analyzed with the 54 targets by metascape (Figure 6c,d). The primary GO BP included the cellular response to nitrogen compound (GO:1901699), modulation of chemical synaptic transmission (GO:0050804), regulation of ion transport (GO:0043269), positive regulation of cell death (GO:0010942), regulation of secretion by cell (GO:1903530), positive regulation of kinase activity (GO:0033674), and synaptic signaling (GO:0099536). Among them, the cellular response to nitrogen compound (GO:1901699) had the highest number of target connections (degree = 20), which included AGER, AGT, CACNA1A, CAV1, CDK5, CHRM2, CHRNB2, GSK3B, TR1A, HTR1B, HTR4, ICAM1, MMP2, MMP3, NTRK2, P2RX7, PKD2, STAT3, CDK5R1, BACE1, CXCL12, ADORA2B, and MERTK. The KEGG pathway showed that the targets significantly correlated with the Alzheimer disease pathway (hsa05010) and neuroactive ligand-receptor interaction (hsa04080). AGER, CACNA1C, CASP8, CDK5, GRIN1, GRIN2B, GSK3B, LPL, MAPT, MME, NOS2, ADAM17, TNFRSF1A, CDK5R1, BACE1, BACE2, UCHL1, and PINK1 were found to be enriched in the AD pathway. We found that AR, CAV1, ICAM1, MMP2, TNFRSF1A, AGT, CASP8, CCR5, and CXCL12 were significantly up-regulated in AD patients compared with controls, while CDK5, GRIN, MAPT, BACE1, GRIN2B, and GRIN3B were significantly down-regulated in AD patients compared with controls. These findings were obtained from the “differential expression” module of the AlzData database (Figure 7). These results demonstrated the potential efficacy of GT in treating AD through its targets in the Aβ and tau pathology.

### 2.2. Cell Model Verification

#### 2.2.1. Effects of GT Extract on OA-Treated SH-SY5Y Cells

**Figure 8:**
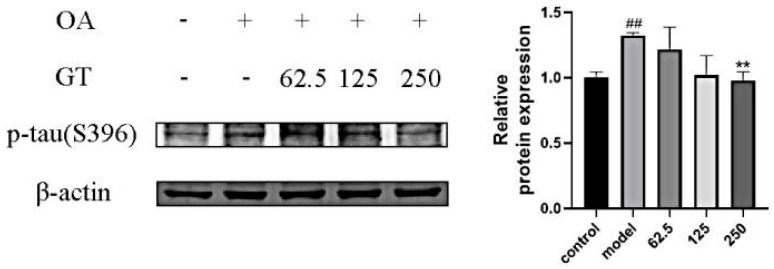
Effect of different concentrations of GT extract (62.5, 125, 250 μg/mL) on tau phosphorylation in OA-induced tau hyperphosphorylation in SHSY-5Y cells. ^##^ *p* < 0.01 compared with control group; ** *p* < 0.01 compared with model group.

Neurofibrillary tangles, a representative pathological change in AD, form due to the aggregation of hyperphosphorylated tau protein. Tau protein, an important microtubule-associated protein, is crucial for maintaining the biological function of microtubules. Therefore, hyperphosphorylation or abnormal expression of tau protein is an important pathological change in AD. In AD, the level to which p-tau increased can induce ROS production [21], and OA can induce ROS generation [22]. In Figure 8, SH-SY5Y cells were simultaneously treated with OA and various concentrations of GT extract for 8 h. The treatment with OA significantly increased the activation of tau phosphorylation in SH-SY5Y cells (*p* < 0.01). GT extract (250 μg/mL) can reverse this increase to some extent (*p* < 0.01). Figure S1 indicates that OA induces ROS generation, and GT can effectively protect against intracellular ROS generation.

#### 2.2.2. The Neuroprotective Effect of GT Extract

Neuronal injury and death, which can be caused by insufficient blood supply, lack of nutrients, and accumulation of metabolites, are relevant in AD. It is believed that oxidative stress plays a crucial role in neurodegenerative diseases such as AD. The pathogenesis of AD has still not been clearly elucidated, but oxidative stress is one of the key hypotheses. Therefore, a H_2_O_2_-induced oxidative damage SH-SY5Y cell model [23,24] and a cell injury model were established by Na_2_S_2_O_4_. Hypoxia stress is a nongenetic inducement of AD [25]. Hence, sodium sulfite was used to induce chemical hypoxia to mimic the hypoxic state of the AD onset.

**Figure 9:**
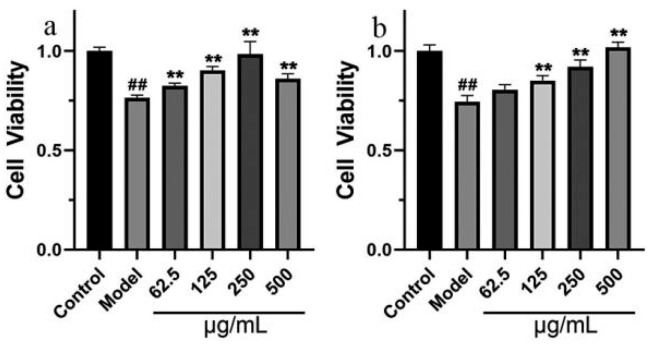
Various concentrations of GT extract suppressed H_2_O_2_-induced cytotoxicity (**a**) and Na_2_S_2_O_4_-induced cytotoxicity (**b**) in SH-SY5Y cells. *^##^ p* < 0.01 compared with control group; ** *p* < 0.01 compared with model group.

The viability of SH-SY5Y cells was measured using MTT assay. The 24 h survival rate of SH-SY5Y cells in the model group, which were damaged by 1 mM H_2_O_2_ or 8 mM Na_2_S_2_O_4_, was significantly lower than that of the control group (*p* < 0.01). Different concentrations of GT extract can reduce the SH-SY5Y cell damage caused by H_2_O_2_ (Figure 9a) or Na_2_S_2_O_4_ (Figure 9b).

## 3. Discussion

AD is a brain disease that results from damage to neurons in the brain [26]. However, the pathogenesis of AD is complex and unclear. Delaying the progression of AD and improving the quality of life of AD patients are important goals for AD treatment. The occurrence and development of the pathological process of AD are related to many factors, the most important of which are Aβ accumulation and tau phosphorylation, which are specific pathophysiological processes of AD [26,27]. Other factors, such as synaptotoxicity, neuroinflammation, oxidative stress, and mitochondrial bioenergetics, have also been reported to be related to the pathological process of AD [28,29].

AD is the most common type of dementia, characterized by the formation of Aβ plaques outside cells and the formation of neuronal fiber tangles (NFTs) inside the cells due to excessive phosphorylation of tau [30]. The deposition of plaques and NFTs is closely related to AD symptoms and can lead to neuronal damage and death.

The behavioral symptoms of AD are directly related to the accumulation of plaques and tangles, which cause lesion of synapses that mediate memory and cognition [31]. This study focused on the effect of GT on p-tau in the pathophysiological process of AD. Tau protein is an important microtubule-related protein and maintains the stability of the microtubule, which is essential to the composition of the neural cytoskeleton [32]. Abnormal post-translational modifications of tau protein mainly manifest as phosphorylation, acetylation, and glycosylation, which can cause neuronal death (ND) and produce symptoms such as cognitive dysfunction [33]. Abnormally hyperphosphorylated tau protein contributes to the formation of neurofibrillary tangles and has cellular neurotoxicity, which is a typical pathological change in the brain of AD patients [34].

TCM involves multiple ingredients and acts on multiple targets and signaling pathways. TCM can relieve symptoms such as insomnia, forgetfulness, disorientation, loss of consciousness, and spasticity, which are associated with the common causes of ND [35]. Research has indicated that the alkaloids of GT are commonly used in the treatment of neurodegenerative diseases, which suggests that GT has potential as a treatment for AD [36,37].

Network pharmacology is a promising research method in drug discovery and can unpack the molecular association between drugs and diseases, unveiling the pharmacological mechanism of drugs in disease treatment at the systemic and biological network level. This provides a new network model for TCM, which targets “multi-target, multi-effect, and complex diseases”, aiding in the prevention and treatment of AD [38].

This study aimed to explore the potential mechanisms, targets, and material basis of GT for preventing and treating AD. A total of 159 related targets for GT and AD were identified, which may be potential targets for treating AD. Some targets were highly relevant in the PPI network and were defined as important targets.

The PPI network analysis of duplicate targets in GT and AD revealed that INS, AKT1, CASP3, EGFR, STAT3, APP, MTOR, MMP9, MAPK1, and PTGS2 were key targets with rich inter-reactions with other targets, indicating a high degree of ability to perform specific molecular functions. Early dysregulation of INS and INSR in AD pathogenesis has also been reported [39]. The oxidative modification of Akt1, mediated by ROS, can cause synaptic dysfunction in AD [40]. AKT1 plays an important role in protecting against Aβ-induced oxidative stress, synaptic/neurotransmission dysfunction, and neurodegeneration [41]. APP is crucial for the initiation and progression of AD by generating Aβ [42,43]. Apoptosis leads to neuronal cell death in various neurodegenerative environments. Activation of CASP3 seems to be a key event in the CNS prior to apoptosis [44]. Additionally, Tesco et al. discovered that CASP3 can cleave the adaptor protein GGA3 in apoptosis, which is required for BACE lysosomal degradation, makes BACE stable, and enhances Aβ generation [45]. Control of STAT3-mediated astrogliosis improves pathology in AD models [46]. The mTOR acts as a crucial effector of cerebrovascular dysregulation in AD [47]. MAPK1 plays an essential role in the cognitive function of rats with AD [48]. The PTGS2 gene is a predisposition gene that relates to the development and progression of AD [49].

The KEGG pathway enrichment analysis showed that the Alzheimer disease pathway (hsa05010, *p* = 9.16 × 10^−39^, degree = 39) was the most important enriched pathway GT for the prevention and treatment. And other pathways such as neuroactive ligand-receptor interaction (hsa04080) and calcium signaling pathway (sa04020) may also relate to GT for the treatment of AD. The “ingredient target-AD target-pathway” network showed that multiple pathways were connected by multiple targets and multiple ingredients, and they worked in conjunction. The effect of GT may relate to corynanthine, angustidine, yohimbine, tetrahydroalstonine, dihydrocorynantheine, hirsutine, hirsuteine, rhynchophylline, isorhynchophylline, and so on. The result of the identification of the ingredients in GT based on UHPLC-ESI-MS/MS analysis proved that the material basis is credible. Corynoxine, corynoxine B, geissoschizine methyl ether, rhynchophylline, and isorhynchophylline can affect the dopaminergic nervous system [50]. Isocorynoxeine, rhynchophylline, isorhynchophylline, and hirsutine can protect against glutamate-induced neuronal death [51]. Rhynchophylline, isorhynchophylline, corynoxeine, and isocorynoxeine can reduce cell damage and heighten cell viability [52,53]. In addition, NMDA inhibitors have been approved as drugs by the FDA for the treatment of cognitive disorders, and memantine hydrochloride is an NMDA inhibitor for the treatment of AD, which has been approved by the FDA. Rhynchophylline was reported to protect against Aβ_1–42_-induced impairment of spatial cognition function by inhibiting excessive activation of extrasynaptic NMDA receptors [54].

Furthermore, analysis of targets bound up in Aβ and tau pathology showed that 54 out of 159 targets were significantly bound up with tau, Aβ, or both. Among them, 21 targets were significantly related to Aβ pathology, and 13 targets were significantly related to tau pathology; 20 were significantly bound up to both Aβ and tau pathology. ICAM1 has a high degree in the PPI network and significantly increases in the entorhinal cortex and temporal cortex of AD patients, which requires further attention. GSK3β and CDK5 kinases are both important kinases among the tau protein kinases and can lead to tau hyperphosphorylation at specific sites under pathological conditions [55,56]. GSK3B is a major tau kinase and constitutes a promising therapeutic target against tauopathies like AD [57]. In addition, the accumulation of GSK3B can promote tau phosphorylation [58].

Oxidative stress has been recognized as a major cause of AD. Oxidative imbalance and subsequent oxidative stress-mediated biomolecular damage has been widely reported in AD, indicating that oxidative damage plays an essential role in AD (Cheignon et al., 2018). Furthermore, it is considered a contributing factor in aging and the progression of various NDs [59]. Additionally, oxidative stress promotes tau hyperphosphorylation [60].

In this study, we mainly focused on the potential effect of GT on reducing tau phosphorylation. We constructed a model induced by OA and found that OA treatment significantly increased the activation of tau phosphorylation in SH-SY5Y cells (*p* < 0.01), and GT extract (250 μg/mL) can reverse this increase to some extent (*p* < 0.01). These results demonstrated that GT can inhibit the activation of p-tau, which provides evidence for treatment with GT in AD. Sodium dithionite and hydrogen peroxide were used to simulate the hypoxic condition and oxidative stress in the pathogenesis of AD, respectively. The results demonstrated the neuroprotective effect of GT against cell damage. These results support further studies on the development of GT as a potential treatment for AD. However, this study also had limitations. In particular, we did not conduct in vivo experiments to demonstrate and clarify the specific mechanism of the effect of GT on AD.

## 4. Materials and Methods

### 4.1. Network Pharmacology

#### 4.1.1. Collection of Active Ingredients and Targets of GT

To identify the active components of GT, the TCM Database and Analysis Platform was searched with the key word “Gou Teng” [61,62] (TCMSP, https://old.tcmsp-e.com/tcmsp.php (accessed on 22 March 2022); TCMID, http://www.megabionet.org/tcmid/ (accessed on 22 March 2022); TCMIP, http://www.tcmip.cn/TCMIP/index.php/ (accessed on 22 March 2022); BATMAN-TCM, http://bionet.ncpsb.org/batman-tcm/ (accessed on 22 March 2022)). The SwissADME system [63] (http://www.swissadme.ch/ (accessed on 22 March 2022)) (Lipinski’s rule of five [64]) was used to identify the candidate ingredients.

#### 4.1.2. Target Prediction

AD target genes were obtained from the databases of DisGeNET (http://www.disgenet.org/ (accessed on 26 March 2022)), GeneCards (https://www.genecards.org/ (accessed on 26 March 2022)), OMIM (https://omim.org/ (accessed on 26 March 2022)), and Therapeutic Target Database (TTD http://db.idrblab.net/ttd/ (accessed on 26 March 2022)) by searching the keyword “Alzheimer’s disease” to obtain related targets [65,66,67]. We adopted the scores from the databases as the evaluation score, screening the top 15% of targets and merging the targets based on manual annotation. The Similarity Ensemble Approach (SEA, http://sea.bkslab.org/ (accessed on 26 March 2022)), Swiss Target Prediction (http://www.swisstargetprediction.ch/ (accessed on 26 March 2022)), TCMSP, and BATMAN-TCM with “homo sapiens” were used to predict the targets of GT [61,68]. Venny 2.1 was used to acquire the related targets between AD and GT. The PPI analysis was acquired by online STRING (https://cn.string-db.org/ (accessed on 26 March 2022)), with interacting genes/proteins and visualized by Cytoscape 3.9.1 software (version 3.9.1, National Institute of General Medical Sciences, San Diego, CA, USA).

#### 4.1.3. Biology Functional Analysis

Metascape (https://metascape.org/ (accessed on 1 April 2022)) was used for GO analysis and KEGG analysis to perform enrichment analysis [69]. Based on the *p*-value, the top 20 results were selected, and we constructed an “ingredients-target-pathway” network using Cytoscape 3.9.1 software.

#### 4.1.4. Molecular Docking

To determine the binding energy between ingredients of GT and targets, we used Autodock Vina and PyMOL for molecular docking. The structures of these targets were collected from the Uniprot Database [67]. Autodock Vina was used for dehydration and hydrogenation. The lower the binding energy, the more stable the docking modules.

#### 4.1.5. Preparation of GT

To prepare the GT extract, 100 g of medicine material was purchased from the Harbin Sankeshu Traditional Chinese Drug Market. It was extracted twice with 70% methanol using the ultrasonic extraction method for 60 min each time. The liquid-solid ratio was 1:10 (*w*/*v*). Then, we filtered the extracting solution and concentrated the extract until it was dry.

#### 4.1.6. UHPLC-Q-Exactive Orbitrap MS Analysis

In order to confirm the presence of bioactive compounds in GT, an Ultimate 3000 UHPLC system (Dionex, Sunnyvale, CA, USA) coupled with a Q-Exactive mass spectrometer (Thermo Fisher Scientific, Waltham, MA, USA) was used to analyze the GT sample. The details are given in the Supplementary Data.

#### 4.1.7. Analysis of GT Targets Related to AD Pathology

The AlzData database [19,20], a high-throughput data collection platform for AD, was utilized to perform correlation analyses of AD pathology (Aβ and tau). All data related to the entorhinal cortex, hippocampus, and temporal cortex were downloaded from the database. The relationship between the potential targets of GT related to AD targets with Aβ and tau pathology was explored.

### 4.2. Pharmacodynamic Experiment

#### 4.2.1. Cell Culture and Treatment

SH-SY5Y cells were seeded in a medium containing 10% FBS and 1% penicillin-streptomycin and cultured at 37 °C with 5% CO_2_. For administration, cells were cultured in 6-well plates for 24 h; then okadaic acid (40 nM OA) and GT extract (62.5, 125, 250 μg/mL) were administered [70]. The ROS was detected by an ROS assay kit with flow cytometry. The cells were first trans-seeded in 96-well plates for 24 h and were then administered with dosing concentrations of 62.5, 125, 250, and 500 μg/mL GT extract. After 2 h, the cells were treated with sodium dithionate (8 mM Na_2_S_2_O_4_) and hydrogen peroxide (1 mM H_2_O_2_) and separately incubated for 24 h. The results were obtained by the MTT method.

#### 4.2.2. Western Blotting Analysis

Cells were sonicated in a RIPA buffer with added proteinase inhibitor (Roche, Basel, Switzerland) and phosphatase inhibitor (APExBIO) (RIPA buffer/phosphatase inhibitor a pipe/phosphatase inhibitor b pipe/proteinase inhibitor 100:1:1:1). The total protein was centrifuged at 12,000 rpm for 15 min at 4 °C, and its concentration was measured using a BCA protein assay kit (Beyotime, Nantong, China). The protein was separated by SDS-PAGE, transferred onto the nitrocellulose membrane, and incubated with primary antibodies overnight at 4 °C (p-tau, Wanleibio Shenyang, China). The membranes were then incubated with fluorescence-conjugated anti-rabbit IgG secondary antibody (Abbkine, Atlanta, GA, USA) for 1 h at room temperature. The protein bands were visualized using the Odyssey Infrared Imaging System (LI-COR, Lincoln, NE, USA).

#### 4.2.3. Statistical Analysis

All statistical analyses were performed via one-way analysis of variance, and the bar plots were generated using GraphPad Prism software (GraphPad Software, San Diego, CA, USA, version 8.0). The data are presented as the mean ± SEM, and statistical significance was set at *p* < 0.05.

## 5. Conclusions

In this study, a link between network pharmacology and in vitro experimental validation was used to explore the potential mechanism of *Uncaria rhynchophylla* for the treatment of AD. The results showed the significant effect of GT in reducing the hyperphosphorylation of tau, and GT can effectively protect against intracellular ROS generation and has neuroprotective effect. This study offers valuable insights into the pathogenesis of AD and the potential mechanism of GT for treating AD.

## Figures and Tables

**Table 1:** Key nodes information in the PPI network.

No.	Name	Uniprot ID	Degree	Betweenness Centrality	Closeness Centrality
1	INS	P01308	95	0.120473547	0.694690265
2	AKT1	P31749	94	0.108167587	0.707207207
3	CASP3	P42574	74	0.024572104	0.633064516
4	EGFR	P00533	72	0.029317310	0.620553360
5	STAT3	P40763	63	0.021227013	0.601532567
6	APP	P05067	62	0.035526890	0.608527132
7	MTOR	P42345	58	0.018850365	0.596958175
8	MMP9	P14780	58	0.013916864	0.579335793
9	ESR1	P03372	57	0.020596430	0.594696970
10	PPARG	P37231	57	0.020462185	0.581481481
11	MAPK1	P28482	57	0.022965376	0.592452830
12	NGF	P01138	57	0.018801898	0.594696970
13	TLR4	O00206	53	0.016064887	0.566787004
14	PTGS2	P35354	52	0.007247996	0.570909091
15	ERBB2	P04626	50	0.010049860	0.564748201
16	LEP	P41159	50	0.014671748	0.572992701
17	CAV1	Q03135	49	0.012189853	0.568840580

**Table 2:** Identified bioactive compounds in GT from UHPLC-ESI-MS/MS Analysis.

ID	Name	Status	Ion Mode
GT2	Akuammigine	Confirmed	Positive
GT3	Angustidine	Confirmed	Positive
GT4	Angustoline	Confirmed	Positive
GT5	Corynantheine	Confirmed	Positive
GT6	Corynanthine	Confirmed	Positive
GT7	Corynoxeine	Confirmed	Positive
GT8	Corynoxine	Confirmed	Positive
GT9	Corynoxine B	Confirmed	Positive
GT10	Dihydrocorynantheine	Confirmed	Positive
GT11	Geissoschizine methyl ether	Confirmed	Positive
GT13	Harman	Confirmed	Positive
GT14	Hirsuteine	Confirmed	Positive
GT15	Hirsutine	Confirmed	Positive
GT16	Isocorynoxeine	Confirmed	Positive
GT18	Isopteropodine	Confirmed	Positive
GT19	Isorhynchophyllic acid	Confirmed	Positive
GT20	Uncarine C	Confirmed	Positive
GT21	Rhynchophylline	Confirmed	Positive
GT22	Tetrahydroalstonine	Confirmed	Positive
GT23	Vallesiachotamine	Confirmed	Positive
GT24	Isorhynchophylline	Confirmed	Positive
GT25	Isomitraphylline	Confirmed	Positive
GT26	Mitraphylline	Confirmed	Positive
GT27	Speciophylline/uncarine D	Confirmed	Positive
GT28	Uncarine F	Confirmed	Positive
GT29	Geissoschizinc acid	Confirmed	Positive
GT30	Rhynchophylline A	Confirmed	Positive
GT31	Methyl-(E)-2-[(2S,3Z,12bS)-3-ethylidene-2,4,6,7,12,12b-hexahydro-1H-indolo [3,2-h]quinolizin-2-yl]-3-methoxyprop-2-enoate	Confirmed	Positive
GT33	Mitraphyllic acid	Confirmed	Positive
GT35	(1′R,3S,4a’S,5a’S,10a’R)-1′-Methyl-2-oxo-1′,4a’,5′,5a’,7′,8′,10′,10a’-octahydrospiro [indoline-3,6′-pyrano [3,4-f]indolizine]-4′-carboxylic acid	Confirmed	Positive
GT36	(2S,12bR)-Methyl-2-((E)-1-oxobut-2-en-2-yl)-1,2,6,7,12,12b-hexahydroindolo [2,3-a]quinolizine-3-carboxylate	Confirmed	Positive
GT37	Vincoside lactam_qt	Confirmed	Positive
GT40	Yohimbin	Confirmed	Positive
GT41	Isocorynantheic acid	Confirmed	Positive
GT42	β-Yohimbin	Confirmed	Positive

## Data Availability

All data generated or analyzed during this study are included in this published article and its supplementary information files.

## Supplementary Materials

The following supporting information can be downloaded at: https://www.mdpi.com/article/10.3390/ijms242015457/s1.

## Abbreviations

Alzheimer’s disease	(AD)
Biological process	(BP)
Cellular component	(CC)
Central nervous system	(CNS)
Food and Drug Administration	(FDA)
Molecular function	(MF)
Neuronal deaths	(NDs)
Neuronal fiber tangles	(NFTs)
Okadaic acid	(OA)
Protein-protein interaction	(PPI)
Traditional Chinese medicine	(TCM)
Ultra-high-performance liquid chromatography	(UHPLC)
*Uncaria rhynchophylla*	(GT)
