# Supplementary material for: Identification of Uncaria rhynchophylla in the Potential Treatment of Alzheimer’s Disease by Integrating Virtual Screening and In Vitro Validation
Source: Int J Mol Sci. 2023 Oct 22;24(20):15457. doi: 10.3390/ijms242015457 (PMC10607254; doi:10.3390/ijms242015457)
Supplement: Supplementary file 1 [file ijms-24-15457-s001.zip › ijms-2615478-Supplementary.pdf]

Identification of *Uncaria rhynchophylla* in the Potential Treatment of Alzheimer's Disease by Integrating Virtual Screening and In Vitro Validation

Shuang Jiang [1,†], Gilwa Borjigin [1,†], Jiahui Sun [1], Qi Li [1], Qianbo Wang [1], Yuanqiu Mu [1], Xuepeng Shi [1], Qian Li [1], Xiaotong Wang [1], Xiaodan Song [1], Zhibin Wang [2] and Chunjuan Yang [1,3,*]

[1] Department of Pharmaceutical Analysis and Analytical Chemistry, College of Pharmacy, Harbin Medical University, Harbin 150081, China
[2] Key Laboratory of Chinese Materia Medica, Ministry of Education, Heilongjiang University of Chinese Medicine, Harbin 150040, China
[3] Key Laboratory of Gut Microbiota and Pharmacogenomics of Heilongjiang Province, College of Pharmacy, Harbin Medical University, Harbin 150081, China
* Correspondence: chunjuanyang@hrbmu.edu.cn
† These authors share the first authorship.

1. Supplementary Information

1.1. Materials and Reagents

Human neuroblastoma SH-SY5Y cells were provided by the College of Pharmacy, Harbin Medical University, (Harbin, China). MTT, hydrogen peroxide (H_2O_2), sodium dithionate ($Na_2S_2O_4$), okadaic acid (OA), dulbecco's modified eagle medium (DMEM) and fetal bovine serum (FBS) were bought from Heilongjiang Jiufeng Bioengineering Co., Ltd (Beijing, China). GT was obtained from the Sankeshu Traditional Chinese Medicine Market in Harbin China.

1.2. UHPLC-Q-Exactive Orbitrap MS Analysis

Chromatographic separation was performed using a Waters ACQUITY UPLC BEH C18 column (100 mm x 2.1 mm, 1.7μm) at 40°C. The mobile phase consisted of water with 0.1% formic acid (A) and acetonitrile (B) at a flow rate of 0.2 mL/min, with the following gradient: 0-3.0 min, 5%B; 3.0-35.0 min, 525%B; 33.0-43.0 min, 2540%B; 43.0-53.0 min, 40100%B ; 53.0-57.0 min, 100%B; 57.0-57.2 min, 1005%B; 57.2-60.0 min, 5%B. The injection volume was 5 μL. After optimizing the ESI conditions, setting were as follows: the sheath gas pressure was 40 arb for positive ion mode and 38 arb for negative ion mode, and the spray voltage was 3.5 kV in positive ion mode and 2.8 kV in negative ion mode. The auxiliary gas flow rate was set to 10 arb, the capillary temperature at 320°C, and the ion source temperature at 300°C for both modes. The scan mode was operated at full-MS with scan ranges of 80-1200 *m*/z and a resolution of 70000. The resolution of the dd-MS2 was set to 17500, the energy gradient is 20/40/60.

2. Supplementary Tables

Table S1. The list of the detected 42 bioactivities.

No.	Compounds	CAS	Structure	Formula	M_W
GT1	akuammicine	639-43-0	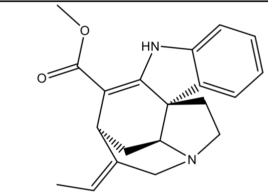	$C_{20}H_{22}N_2O_2$	322.4
GT2	akuammigine	642-17-1	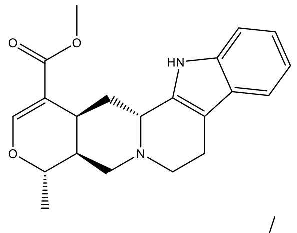	$C_{21}H_{24}N_2O_3$	352.4
GT3	angustidine	40217-50-3	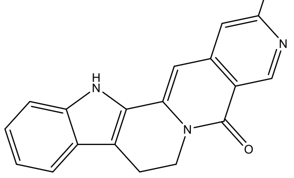	$C_{19}H_{15}N_3O$	301.3
GT4	angustoline	40041-95-0	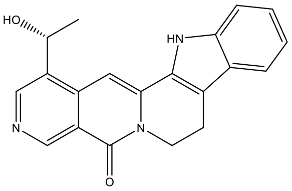	$C_{20}H_{17}N_3O_2$	331.4
GT5	corynantheine	18904-54-6	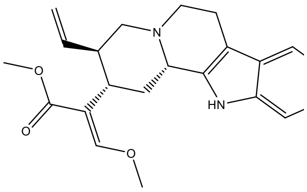	$C_{22}H_{26}N_2O_3$	366.5
GT6	corynanthine	483-10-3	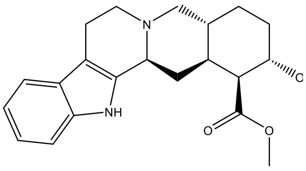	$C_{21}H_{26}N_2O_3$	354.4
GT7	corynoxeine	630-94-4	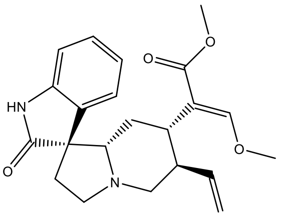	$C_{22}H_{26}N_2O_4$	382.5
GT8	corynoxine	6877-32-3	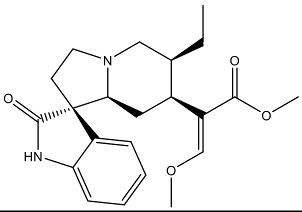	$C_{22}H_{28}N_2O_4$	384.5

GT9	corynoxine b	17391-18-3	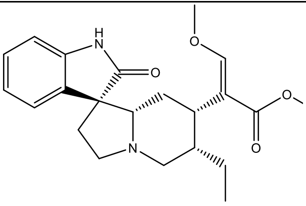	$C_{22}H_{28}N_2O_4$	384.5
GT10	dihydrocorynantheine	50439-68-4	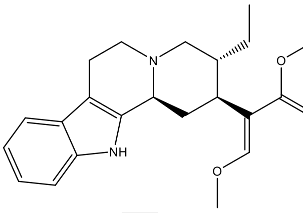	$C_{22}H_{28}N_2O_3$	368.5
GT11	Geissoschizine Methyl Ether	60314-89-8	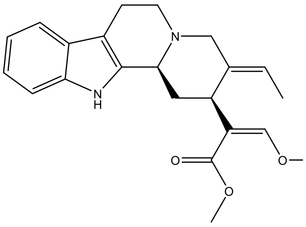	$C_{22}H_{26}N_2O_3$	366.5
GT12	gelsamydine	120881-61-0	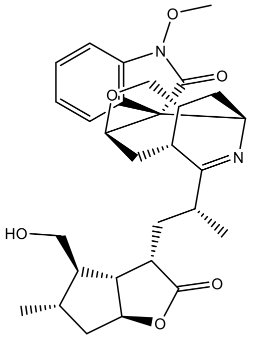	$C_{29}H_{36}N_2O_6$	508.6
GT13	harman	486-84-0	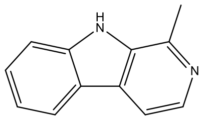	$C_{12}H_{10}N_2$	182.2
GT14	hirsuteine	35467-43-7	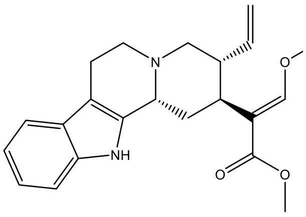	$C_{22}H_{26}N_2O_3$	366.5
GT15	hirsutine	7729-23-9	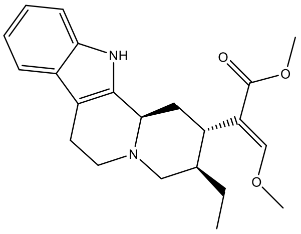	$C_{22}H_{28}N_2O_3$	368.5
GT16	isocorynoxeine	51014-29-0	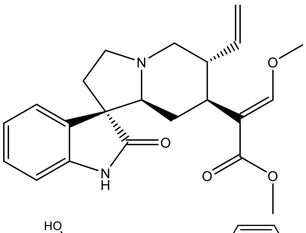	$C_{22}H_{26}N_2O_4$	382.5
GT17	isocorypalmine	483-34-1	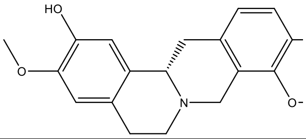	$C_{20}H_{23}NO_4$	341.4

GT18	isopteropodine	5171-37-9	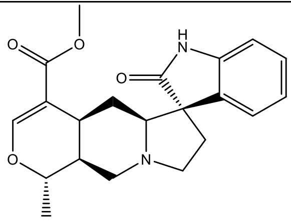	$C_{21}H_{24}N_2O_4$	368.4
GT19	Isorhynchophylline	144525-05-3	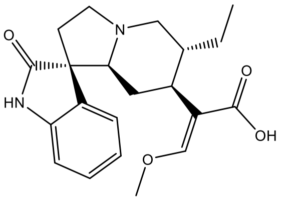	$C_{22}H_{28}N_2O_4$	370.5
GT20	pteropodine/Uncarine C	5629-60-7	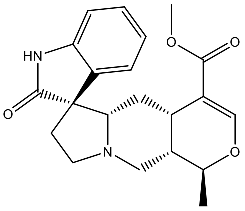	$C_{21}H_{24}N_2O_4$	368.4
GT21	rhynchophylline	76-66-4	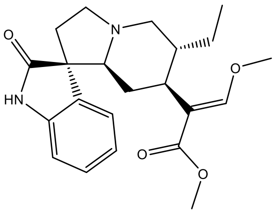	$C_{22}H_{28}N_2O_4$	384.5
GT22	tetrahydroalstonine	6474-90-4	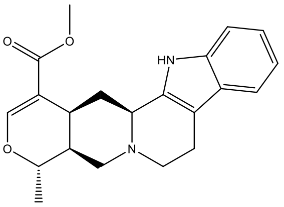	$C_{21}H_{24}N_2O_3$	352.4
GT23	vallesiachotamine	5523-37-5	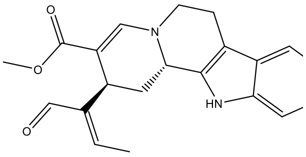	$C_{21}H_{22}N_2O_3$	350.4
GT24	Isorhyncophylline	6859-01-4	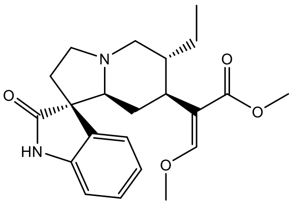	$C_{26}H_{30}N_2O_8$	498.5
GT25	Isomitraphylline	4963-01-3	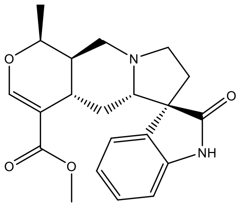	$C_{21}H_{24}N_2O_4$	368.4
GT26	Mitraphylline	509-80-8	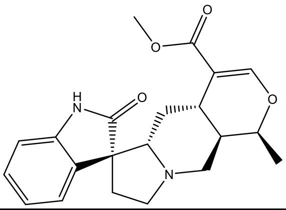	$C_{21}H_{24}N_2O_4$	368.4

GT27	Uncarine D	4697-68-1	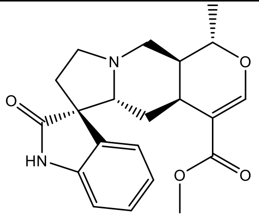	$C_{21}H_{24}N_2O_4$	368.4
GT28	Uncarine F	14019-66-0	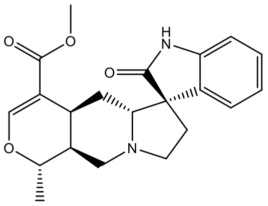	$C_{21}H_{24}N_2O_4$	368.4
GT29	geissoschizinc acid		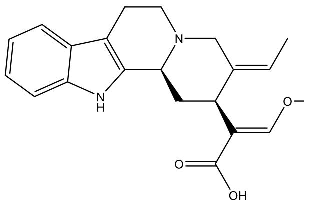	$C_{21}H_{24}N_2O_3$	352.4
GT30	Rhynchophylline A		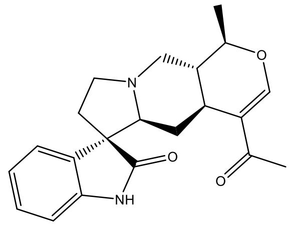	$C_{21}H_{24}N_2O_3$	352.4
GT31	methyl (E)-2-[(2S,3Z,12bS)-3-ethylidene-2,4,6,7,12,12b-hexahydro-1H-indolo[3,2-h]quinolizin-2-yl]-3-methoxyprop-2-enoate		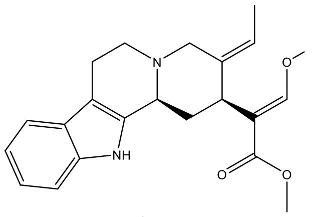	$C_{22}H_{26}N_2O_3$	366.5
GT32	hirsutasideA		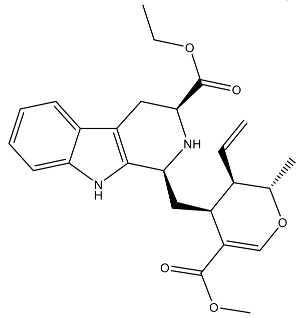	$C_{25}H_{30}N_2O_5$	438.6
GT33	Mitraphyllic acid	10126-00-8	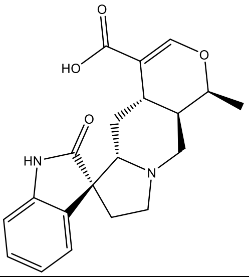	$C_{20}H_{22}N_2O_4$	354.4

GT34	Hirsutaside B		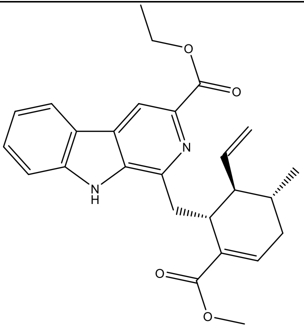	$C_{26}H_{28}N_2O_4$	432.6
GT35	(1'R,3S,4a'S,5a'S,10a'R)-1'-methyl-2-oxo-1',4a',5',5a',7',8',10',10a'-octahydrospiro[indoline-3,6'-pyrano[3,4-f]indolizine]-4'-carboxylic acid		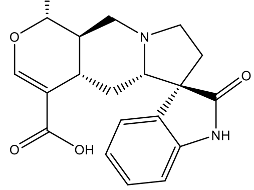	$C_{20}H_{22}N_2O_4$	354.4
GT36	(2S,12bR)-methyl 2-((E)-1-oxobut-2-en-2-yl)-1,2,6,7,12,12b-hexahydroindolo[2,3-a]quinolizine-3-carboxylate		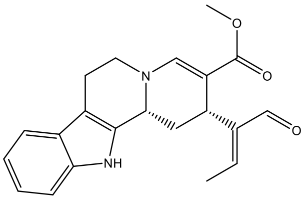	$C_{21}H_{22}N_2O_3$	350.5
GT37	vincoside lactam_qt		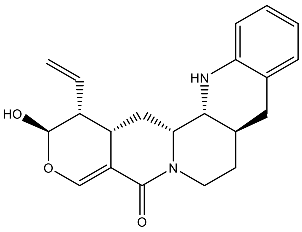	$C_{21}H_{24}N_2O_3$	352.5
GT38	hirsutasideC		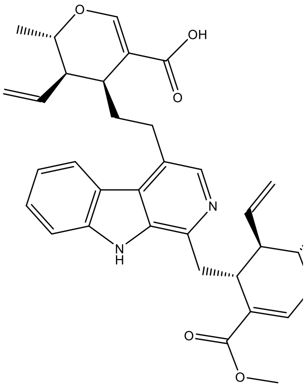	$C_{33}H_{36}N_2O_6$	556.7
GT39	Isorhynchophyllic acid		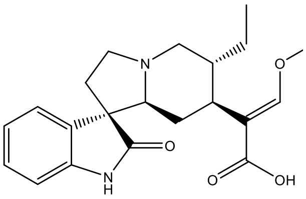	$C_{21}H_{26}N_2O_4$	370.4
GT40	Yohimbine	146-48-5	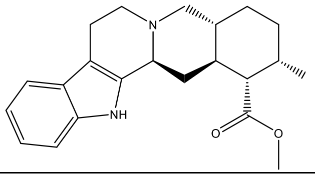	$C_{21}H_{26}N_2O_3$	354.4

GT41	isocorynantheic acid		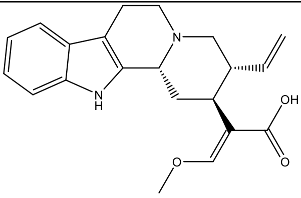	$C_{21}H_{24}N_2O_3$	352.5
GT42	coryincine/beta-Yohimbine	549-84-8	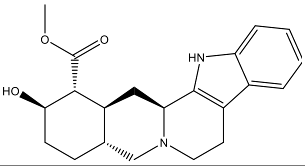	$C_{21}H_{26}N_2O_3$	354.4

Table S2. Physicochemical properties of bioactivities for Lipinski's rule, bioavailability, and cell membrane permeability.

No.	Compounds	Lipinski's rule				Lipinski's Violations	Bioavailability Score	TPSA (Å^2)
		MW	HBA	HBD	MLogP			
		<500	<10	≤5	≤4.15	≤1	>0.1	<140
GT1	akuammicine	322.4	3	1	2.82	0	0.55	41.57
GT2	akuammigine	352.4	3	1	2.82	0	0.55	41.57
GT3	angustidine	301.3	2	1	2.44	0	0.55	50.68
GT4	angustoline	331.4	3	2	1.84	0	0.55	70.91
GT5	corynantheine	366.5	4	1	2.27	0	0.85	54.56
GT6	corynanthine	354.4	4	2	2.21	0	0.55	65.56
GT7	corynoxeine	382.5	5	1	1.94	0	0.85	67.87
GT8	corynoxine	384.5	5	1	2.02	0	0.85	67.87
GT9	corynoxine b	384.5	5	1	2.02	0	0.85	67.87
GT10	dihydrocorynantheine	368.5	4	1	2.35	0	0.85	54.56
GT11	Geissoschizine Methyl Ether	366.5	4	1	2.27	0	0.85	54.56
GT12	gelsamydine	508.6	7	1	2.68	1	0.55	97.66
GT13	harman	182.2	1	1	1.9	0	0.55	28.68
GT14	hirsuteine	366.5	4	1	2.27	0	0.85	54.56
GT15	hirsutine	368.5	4	1	2.35	0	0.85	54.56
GT16	isocorynoxeine	382.5	5	1	1.94	0	0.85	67.87
GT17	isocorypalmine	341.4	5	1	1.98	0	0.55	51.16
GT18	isopteropodine	368.4	5	1	1.8	0	0.85	67.87
GT19	isorhynchophylline	370.5	5	2	1.8	0	0.56	78.87
GT20	pteropodine/Uncarine C	368.4	5	1	1.8	0	0.85	67.87
GT21	rhynchophylline	384.5	5	1	2.02	0	0.85	67.87
GT22	tetrahydroalstonine	352.4	4	1	2.13	0	0.85	54.56
GT23	vallesiachotamine	350.4	3	1	1.98	0	0.55	62.40
GT24	Isorhyncophylline	498.5	5	1	2.02	0	0.85	67.87
GT25	Isomitraphylline	368.4	5	1	1.8	0	0.85	67.87
GT26	Mitraphylline	368.4	5	1	1.8	0	0.85	67.87
GT27	Uncarine D	368.4	5	1	1.8	0	0.85	67.87
GT28	Uncarine F	368.4	5	1	1.8	0	0.85	67.87
GT29	geissoschizinc acid	352.4	7	3	-	-	-	107.13
GT30	Rhynchophylline A	352.4	4	1	1.8	0	0.55	58.64
GT31	methyl (E)-2-[(2S,3Z,12bS)-3-ethylidene-2,4,6,7,12,12b-hexahydro-1H-indolo[3,2-h]quinolizin-2-yl]-3-methoxyprop-2-enoate	366.5	4	1	-	-	-	54.56
GT32	hirsutasideA	438.6	6	2	2.03	0	0.55	89.65
GT33	Mitraphyllic acid	354.4	5	2	1.58	0	0.56	78.87
GT34	hirsutasideB	432.6	5	1	3.39	0	0.55	81.28
GT35	(1'R,3S,4a'S,5a'S,10a'R)-1'-methyl-2-oxo-	354.4	5	2	1.39	0	0.56	78.87

	1',4a',5',5a',7',8',10',10a'-octahydrospiro[indoline-3,6'-pyrano[3,4-f]indolizine]-4'-carboxylic acid							
GT36	(2S,12bR)-methyl 2-((E)-1-oxobut-2-en-2-yl)-1,2,6,7,12,12b-hexahydroindolo[2,3-a]quinolizine-3-carboxylate	350.5	3	1	1.98	0	0.55	62.40
GT37	vincoside lactam_qt	352.5	3	2	2.21	0	0.85	61.80
GT38	hirsutasideC	556.7	7	2	2.74	1	0.56	110.74
GT39	Isorhynchophyllic acid	370.4	5	2	1.8	0	0.56	78.87
GT40	Yohimbine	354.4	4	2	2.21	0	0.55	65.56
GT41	isocorynantheic acid	352.5	4	2	2.05	0	0.85	65.56
GT42	coryincine/beta-Yohimbine	354.4	4	2	2.21	0	0.55	65.56

Table S3. The connection information of shared genes.

No.	Uniprot ID	Targets	Description
1	Q01959	SLC6A3	Sodium-dependent dopamine transporter
2	P20309	CHRM3	Muscarinic acetylcholine receptor M3
3	Q13224	GRIN2B	Glutamate receptor ionotropic, NMDA 2B
4	Q05586	GRIN1	Glutamate receptor ionotropic, NMDA 1
5	P11229	CHRM1	Muscarinic acetylcholine receptor M1
6	P08912	CHRM5	Muscarinic acetylcholine receptor M5
7	P31645	SLC6A4	Sodium-dependent serotonin transporter
8	P08172	CHRM2	Muscarinic acetylcholine receptor M2
9	P08908	HTR1A	5-hydroxytryptamine receptor 1A
10	P14416	DRD2	D(2) dopamine receptor
11	P21917	DRD4	D(4) dopamine receptor
12	P50406	HTR6	5-hydroxytryptamine receptor 6
13	P28223	HTR2A	5-hydroxytryptamine receptor 2A
14	P28335	HTR2C	5-hydroxytryptamine receptor 2C
15	P28222	HTR1B	5-hydroxytryptamine receptor 1B
16	P43681	CHRNA4	Neuronal acetylcholine receptor subunit alpha-4
17	P35462	DRD3	D(3) dopamine receptor
18	O60674	JAK2	Tyrosine-protein kinase JAK2
19	P08183	ABCB1	ATP-dependent translocase ABCB1
20	P27338	MAOB	Amine oxidase [flavin-containing] B
21	P10635	CYP2D6	Cytochrome P450 2D6
22	Q05940	SLC18A2	Synaptic vesicular amine transporter
23	P36544	CHRNA7	Neuronal acetylcholine receptor subunit alpha-7
24	P06276	BCHE	Cholinesterase
25	P29475	NOS1	Nitric oxide synthase, brain
26	Q13639	HTR4	5-hydroxytryptamine receptor 4
27	P35228	NOS2	Nitric oxide synthase, inducible
28	P28845	HSD11B1	11-beta-hydroxysteroid dehydrogenase 1
29	Q5S007	LRRK2	Leucine-rich repeat serine/threonine-protein kinase 2
30	P17787	CHRNB2	Neuronal acetylcholine receptor subunit beta-2
31	P30926	CHRNB4	Neuronal acetylcholine receptor subunit beta-4
32	P18825	ADRA2C	Alpha-2C adrenergic receptor
33	P40763	STAT3	Signal transducer and activator of transcription 3
34	P28221	HTR1D	5-hydroxytryptamine receptor 1D
35	P12821	ACE	Angiotensin-converting enzyme
36	Q16539	MAPK14	Mitogen-activated protein kinase 14
37	P07711	CTSL	Procathepsin L
38	Q99572	P2RX7	P2X purinoceptor 7
39	P29474	NOS3	Nitric oxide synthase, endothelial
40	P00533	EGFR	Epidermal growth factor receptor

41	P49841	GSK3B	Glycogen synthase kinase-3 beta
42	Q15078	CDK5R1	Cyclin-dependent kinase 5 activator 1
43	Q13627	DYRK1A	Dual specificity tyrosine-phosphorylation-regulated kinase 1A
44	P31749	AKT1	RAC-alpha serine/threonine-protein kinase
45	P31644	GABRA5	Gamma-aminobutyric acid receptor subunit alpha-5
46	P08581	MET	Hepatocyte growth factor receptor
47	P07333	CSF1R	Macrophage colony-stimulating factor 1 receptor
48	P00734	F2	Prothrombin
49	Q00535	CDK5	Cyclin-dependent-like kinase 5
50	P35354	PTGS2	Prostaglandin G/H synthase 2
51	P30556	AGTR1	Type-1 angiotensin II receptor
52	P32245	MC4R	Melanocortin receptor 4
53	P07949	RET	Proto-oncogene tyrosine-protein kinase receptor Ret
54	P04179	SOD2	Superoxide dismutase [Mn], mitochondrial
55	P37840	SNCA	Alpha-synuclein
56	P00441	SOD1	Superoxide dismutase [Cu-Zn]
57	Q13563	PKD2	Polycystin-2
58	P10415	BCL2	Apoptosis regulator Bcl-2
59	P01019	AGT	Angiotensinogen
60	P16671	CD36	Platelet glycoprotein 4
61	P01308	INS	Insulin
62	Q15109	AGER	Advanced glycosylation end product-specific receptor
63	P21964	COMT	Catechol O-methyltransferase
64	P00797	REN	Renin
65	P49810	PSEN2	Presenilin-2
66	P42892	ECE1	Endothelin-converting enzyme 1
67	P06213	INSR	Insulin receptor
68	P22607	FGFR3	Fibroblast growth factor receptor 3
69	P04629	NTRK1	High affinity nerve growth factor receptor
70	Q16620	NTRK2	BDNF/NT-3 growth factors receptor
71	P04626	ERBB2	Receptor tyrosine-protein kinase erbB-2
72	P11362	FGFR1	Fibroblast growth factor receptor 1
73	P08473	MME	Neprilysin
74	Q13093	PLA2G7	Platelet-activating factor acetylhydrolase
75	Q9NZ42	PSENEN	Gamma-secretase subunit PEN-2
76	Q92542	NCSTN	Nicastrin
77	P49768	PSEN1	Presenilin-1
78	P10275	AR	Androgen receptor
79	P05023	ATP1A1	Sodium/potassium-transporting ATPase subunit alpha-1
80	P78536	ADAM17	Disintegrin and metalloproteinase domain-containing protein 17
81	P06737	PYGL	Glycogen phosphorylase, liver form
82	P51681	CCR5	C-C chemokine receptor type 5
83	Q12866	MERTK	Tyrosine-protein kinase Mer
84	P14780	MMP9	Matrix metalloproteinase-9
85	Q08499	PDE4D	cAMP-specific 3',5'-cyclic phosphodiesterase 4D
86	P56817	BACE1	Beta-secretase 1
87	P48039	MTNR1A	Melatonin receptor type 1A
88	P42574	CASP3	Caspase-3
89	Q14790	CASP8	Caspase-8
90	P37231	PPARG	Peroxisome proliferator-activated receptor gamma
91	Q13526	PIN1	Peptidyl-prolyl cis-trans isomerase NIMA-interacting 1
92	O14746	TERT	Telomerase reverse transcriptase
93	P02753	RBP4	Retinol-binding protein 4
94	P11511	CYP19A1	Aromatase
95	P41159	LEP	Leptin
96	O60260	PARK2	E3 ubiquitin-protein ligase parkin
97	P21333	FLNA	Filamin-A

98	O00555	CACNA1A	Voltage-dependent P/Q-type calcium channel subunit alpha-1A
99	Q13936	CACNA1C	Voltage-dependent L-type calcium channel subunit alpha-1C
100	P06850	CRH	Corticoliberin
101	P29274	ADORA2A	Adenosine receptor A2a
102	P29275	ADORA2B	Adenosine receptor A2b
103	P21802	FGFR2	Fibroblast growth factor receptor 2
104	Q03135	CAV1	Caveolin-1
105	P09936	UCHL1	Ubiquitin carboxyl-terminal hydrolase isozyme L1
106	Q9BXM7	PINK1	Serine/threonine-protein kinase PINK1, mitochondrial
107	P30872	SSTR1	Somatostatin receptor type 1
108	P31391	SSTR4	Somatostatin receptor type 4
109	P30874	SSTR2	Somatostatin receptor type 2
110	P32745	SSTR3	Somatostatin receptor type 3
111	Q9HCR9	PDE11A	Dual 3',5'-cyclic-AMP and -GMP phosphodiesterase 11A
112	P35346	SSTR5	Somatostatin receptor type 5
113	O00206	TLR4	Toll-like receptor 4
114	P34969	HTR7	5-hydroxytryptamine receptor 7
115	P37173	TGFBR2	TGF-beta receptor type-2
116	P42336	PIK3CA	Phosphatidylinositol 4,5-bisphosphate 3-kinase catalytic subunit alpha isoform
117	P05362	ICAM1	Intercellular adhesion molecule 1
118	P49238	CX3CR1	CX3C chemokine receptor 1
119	P09619	PDGFRB	Platelet-derived growth factor receptor beta
120	P41597	CCR2	C-C chemokine receptor type 2
121	Q13315	ATM	Serine-protein kinase ATM
122	P42345	MTOR	Serine/threonine-protein kinase mTOR
123	P10721	KIT	Mast/stem cell growth factor receptor Kit
124	O95263	PDE8B	High affinity cAMP-specific and IBMX-insensitive 3',5'-cyclic phosphodiesterase 8B
125	O60760	HPGDS	Hematopoietic prostaglandin D synthase
126	P11473	VDR	Vitamin D3 receptor
127	P04049	RAF1	RAF proto-oncogene serine/threonine-protein kinase
128	P08253	MMP2	72 kDa type IV collagenase
129	P07339	CTSD	Cathepsin D
130	P04035	HMGCR	3-hydroxy-3-methylglutaryl-coenzyme A reductase
131	O14684	PTGES	Prostaglandin E synthase
132	Q9Y5Z0	BACE2	Beta-secretase 2
133	P08254	MMP3	Stromelysin-1
134	P03956	MMP1	Interstitial collagenase
135	P41180	CASR	Extracellular calcium-sensing receptor
136	P07858	CTSB	Cathepsin B
137	P17655	CAPN2	Calpain-2 catalytic subunit
138	P35968	KDR	Vascular endothelial growth factor receptor 2
139	P28482	MAPK1	Mitogen-activated protein kinase 1
140	P42858	HTT	Huntingtin
141	O60885	BRD4	Bromodomain-containing protein 4
142	P14174	MIF	Macrophage migration inhibitory factor
143	P15056	BRAF	Serine/threonine-protein kinase B-raf
144	P02766	TTR	Transthyretin
145	P03372	ESR1	Estrogen receptor
146	Q92731	ESR2	Estrogen receptor beta
147	P07101	TH	Tyrosine 3-monooxygenase
148	P05164	MPO	Myeloperoxidase
149	P10636	MAPT	Microtubule-associated protein tau
150	P48061	CXCL12	Stromal cell-derived factor 1
151	Q13546	RIPK1	Receptor-interacting serine/threonine-protein kinase 1

152	Q07869	PPARA	Peroxisome proliferator-activated receptor alpha
153	Q02156	PRKCE	Protein kinase C epsilon type
154	P19438	TNFRSF1A	Tumor necrosis factor receptor superfamily member 1A
155	P13807	GYS1	Glycogen [starch] synthase, muscle
156	P14735	IDE	Insulin-degrading enzyme
157	P05067	APP	Amyloid-beta precursor protein
158	P01138	NGF	Beta-nerve growth factor
159	P06858	LPL	Lipoprotein lipase

Table S4. Targets in 20 signaling pathways' enrichment related to Alzheimer disease.

KEGG ID	Description	Count	Targets	Log (q-value)
hsa05010	Alzheimer disease	39	AGER,AKT1,APP,BRAF,CACNA1C,CAPN2,CASP3, CASP8,CDK5,CHRM1,CHRM3,CHRM5,CHRNA7, MTOR,GRIN1,GRIN2B,GSK3B,IDE,INS,INSR,LPL, MAPT,MME,NOS1,NOS2,PIK3CA,MAPK1,PSEN1, PSEN2,PTGS2,RAF1,SNCA,ADAM17,TNFRSF1A, CDK5R1,NCSTN,BACE1,BACE2,PSENEN	-35.5
hsa04080	Neuroactive ligand-receptor interaction	38	ADORA2A,ADORA2B,ADRA2C,AGT,AGTR1,CHRM1,CHRM2,CHRM3,CHRM5,CHRNA4,CHRNA7,CHRNB2,CHRNB4,CRH,DRD2,DRD3,DRD4,F2,GABRA5,GRIN1,GRIN2B,HTR1A,HTR1B,HTR1D,HTR2A,HTR2C,HTR4,HTR6,HTR7,LEP,MC4R,MTNR1A,P2RX7,SSTR1,SSTR2,SSTR3,SSTR4,SSTR5	-35.35
hsa04020	Calcium signaling pathway	32	ADORA2A,ADORA2B,AGTR1,CACNA1A,CACNA1C,CHRM1,CHRM2,CHRM3,CHRM5,CHRNA7,EGFR,ERBB2,FGFR1,FGFR3,FGFR2,GRIN1,HTR2A,HTR2C,HTR4,HTR6,HTR7,KDR,MET,NGF,NOS1,NOS2,NOS3,NTRK1,NTRK2,P2RX7,PDGFRB,RET	-32.86
hsa05200	Pathways in cancer	38	AGT,AGTR1,AKT1,AR,BCL2,BRAF,CASP3,CASP8, CSF1R,EGFR,ERBB2,ESR1,ESR2,F2,FGFR1,FGFR3,FGFR2,MTOR,GSK3B,JAK2,KIT,MET,MMP1,MMP2,MMP9,NOS2,NTRK1,PDGFRB,PIK3CA,PPARG,MAPK1,PTGS2,RAF1,RET,CXCL12,STAT3,TERT,TGFBR2	-29.33
hsa04726	Serotonergic synapse	20	APP,BRAF,CACNA1A,CACNA1C,CASP3,CYP2D6,HTR1A,HTR1B,HTR1D,HTR2A,HTR2C,HTR4,HTR6,HTR7,MAOB,MAPK1,PTGS2,RAF1,SLC6A4,SLC18A2	-22.53
hsa01521	EGFR tyrosine kinase inhibitor resistance	17	AKT1,BCL2,BRAF,EGFR,ERBB2,FGFR3,FGFR2,MTOR,GSK3B,JAK2,KDR,MET,PDGFRB,PIK3CA,MAPK1,RAF1,STAT3	-20.84
hsa05417	Lipid and atherosclerosis	21	AGER,AKT1,BCL2,CASP3,CASP8,CD36,MAPK14, GSK3B,ICAM1,JAK2,MMP1,MMP3,MMP9,NOS3,PIK3CA,PPARG,MAPK1,SOD2,STAT3,TLR4,TNFRSF1A	-18.59
hsa05415	Diabetic cardiomyopathy	20	AGER,AGT,AGTR1,AKT1,CD36,MAPK14,CTSD,ACE,MTOR,GSK3B,GYS1,INS,INSR,MMP2,MMP9,NOS3,PIK3CA,PPARA,REN,TGFBR2	-17.81
hsa05207	Chemical carcinogenesis - receptor activation	20	AKT1,AR,BCL2,CACNA1A,CACNA1C,CHRNA4,CHRNA7,CHRNB2,CHRNB4,EGFR,ESR1,ESR2,MTOR,JAK2,PIK3CA,PPARA,MAPK1,RAF1,STAT3,VDR	-17.48
hsa04728	Dopaminergic synapse	14	AKT1,CACNA1A,CACNA1C,COMT,MAPK14,DRD2,DRD3,DRD4,GRIN2B,GSK3B,MAOB,SLC6A3,SLC18A2,TH	-12.84
hsa05418	Fluid shear stress and	12	AKT1,BCL2,CAV1,MAPK14,CTSL,ICAM1,KDR,MMP2,MMP9,NOS3,PIK3CA,TNFRSF1A	-10.13

	atherosclerosis			
hsa05171	Coronavirus disease - COVID-19	13	AGTR1,MAPK14,ACE,EGFR,F2,MMP1,MMP3,PIK3CA,MAPK1,STAT3,ADAM17,TLR4,TNFRSF1A	-8.73
hsa05033	Nicotine addiction	7	CACNA1A,CHRNA4,CHRNA7,CHRNB2,GABRA5,GRIN1,GRIN2B	-8.12
hsa04920	Adipocytokine signaling pathway	8	AKT1,CD36,MTOR,JAK2,LEP,PPARA,STAT3,TNFRSF1A	-7.84
hsa04934	Cushing syndrome	10	AGT,AGTR1,BRAF,CACNA1C,CRH,EGFR,GSK3B,MAPK1,PDE8B,PDE11A	-7.35
hsa04064	NF-kappa B signaling pathway	8	ATM,BCL2,ICAM1,PTGS2,CXCL12,TLR4,TNFRSF1A,RIPK1	-6.5
hsa04520	Adherens junction	7	EGFR,ERBB2,FGFR1,INSR,MET,MAPK1,TGFBR2	-6.43
hsa04540	Gap junction	7	DRD2,EGFR,HTR2A,HTR2C,PDGFRB,MAPK1,RAF1	-5.82
hsa05014	Amyotrophic lateral sclerosis	12	BCL2,CASP3,MAPK14,MTOR,GRIN1,GRIN2B,NOS1,NOS2,PRKN,SOD1,TNFRSF1A,PINK1	-5.71
hsa04621	NOD-like receptor signaling pathway	9	BCL2,CASP8,CASR,MAPK14,CTSB,P2RX7,MAPK1,TLR4,RIPK1	-5.68

Table S5. The related information of components docked with key targets.

No.	Gene	Component	Binding energy (kcal/mol)	No.	Gene	Component	Binding energy (kcal/mol)
1	AKT1	GT2	-7	69	mTOR	GT9	-8.1
2	AKT1	GT5	-6.1	70	mTOR	GT12	-6
3	AKT1	GT6	-6.7	71	mTOR	GT16	-6.3
4	AKT1	GT7	-5.9	72	mTOR	GT17	-6.7
5	AKT1	GT8	-6.2	73	mTOR	GT19	-7.9
6	AKT1	GT10	-6.4	74	mTOR	GT20	-6.8
7	AKT1	GT11	-5.7	75	mTOR	GT21	-6.4
8	AKT1	GT14	-6.1	76	mTOR	GT24	-6.9
9	AKT1	GT15	-6	77	mTOR	GT25	-7.1
10	AKT1	GT18	-6.7	78	mTOR	GT26	-7.1
11	AKT1	GT19	-6.2	79	mTOR	GT27	-8
12	AKT1	GT20	-6.3	80	mTOR	GT28	-7.4
13	AKT1	GT21	-5.9	81	NGF	GT40	-8.1
14	AKT1	GT24	-6	82	PPARG	GT5	-6.6
15	AKT1	GT25	-6.4	83	PPARG	GT14	-7
16	AKT1	GT26	-6.5	84	PPARG	GT15	-8.4
17	AKT1	GT27	-6.6	85	PPARG	GT22	-7.2
18	AKT1	GT28	-6.7	86	PPARG	GT29	-7.1
19	AKT1	GT31	-6.3	87	PPARG	GT31	-7.7
20	AKT1	GT42	-7.1	88	PPARG	GT32	-7.1
21	APP	GT33	-6.3	89	PPARG	GT36	-8.1
22	APP	GT39	-6.1	90	PPARG	GT37	-6.6
23	CASP3	GT4	-7.2	91	PPARG	GT41	-8.7
24	CASP3	GT10	-7.3	92	PPARG	GT40	-8.8
25	CASP3	GT11	-6.7	93	PPARG	GT42	-7
26	CASP3	GT14	-7.1	94	STAT3	GT2	-7.9
27	CASP3	GT40	-6.1	95	STAT3	GT3	-7.6
28	CAV1	GT6	-7	96	STAT3	GT6	-6.8

29	EGFR	GT2	-7.4	97	STAT3	GT22	-7.4
30	EGFR	GT3	-8.2	98	STAT3	GT23	-7.8
31	EGFR	GT4	-8	99	STAT3	GT40	-7.7
32	EGFR	GT7	-6.8	100	STAT3	GT42	-8.5
33	EGFR	GT8	-6.8	101	STAT3	GT12	-7.4
34	EGFR	GT9	-6.8	102	TLR4	GT6	-7.3
35	EGFR	GT11	-6.9	103	TLR4	GT40	-8.1
36	EGFR	GT12	-6.5	104	TLR4	GT42	-7.1
37	EGFR	GT13	-6.8	105	PTGS2	GT7	-8.2
38	EGFR	GT16	-7.4	106	PTGS2	GT8	-9.5
39	EGFR	GT19	-7.6	107	PTGS2	GT12	-7.6
40	EGFR	GT20	-6.8	108	PTGS2	GT13	-8.3
41	EGFR	GT21	-6.9	109	PTGS2	GT14	-8.2
42	EGFR	GT24	-7.1	110	PTGS2	GT15	-8.3
43	EGFR	GT25	-7.5	111	PTGS2	GT16	-8.5
44	EGFR	GT26	-7.2	112	PTGS2	GT17	-8.4
45	EGFR	GT27	-7.4	113	PTGS2	GT18	-7.9
46	EGFR	GT28	-7.6	114	PTGS2	GT20	-7.6
47	EGFR	GT33	-9.3	115	PTGS2	GT21	-7.8
48	ERBB2	GT3	-6.8	116	PTGS2	GT24	-8.2
49	ERBB2	GT4	-7	117	PTGS2	GT27	-7.4
50	ERBB2	GT6	-7.4	118	PTGS2	GT29	-8.3
51	ERBB2	GT20	-6.7	119	PTGS2	GT31	-7.8
52	ERBB2	GT26	-6.3	120	PTGS2	GT33	-8.3
53	ERBB2	GT28	-6.5	121	PTGS2	GT34	-8
54	ERBB2	GT40	-6.4	122	PTGS2	GT36	-7.8
55	ERBB2	GT42	-6.7	123	PTGS2	GT37	-6.5
56	ESR1	GT17	-7.9	124	PTGS2	GT41	-9.6
57	INS	GT3	-7.3	125	PTGS2	GT3	-9.7
58	LEP	GT6	-6.7	126	PTGS2	GT4	-8.6
59	MAPK1	GT12	-8.4	127	PTGS2	GT5	-8.6
60	MMP9	GT4	-8.3	128	PTGS2	GT10	-9
61	MMP9	GT7	-8	129	PTGS2	GT19	-9.1
62	MMP9	GT8	-8.8	130	PTGS2	GT22	-8.6
63	MMP9	GT12	-8.5	131	PTGS2	GT30	-8.6
64	MMP9	GT16	-8.9	132	PTGS2	GT32	-9.1
65	MMP9	GT17	-10.7	133	PTGS2	GT35	-8.7
66	MMP9	GT23	-8.9	134	PTGS2	GT38	-9
67	mTOR	GT7	-6.8	135	PTGS2	GT40	-8.6
68	mTOR	GT8	-6.8	136	PTGS2	GT42	-8.1

Table S6. Peak information of GT by UHPLC-ESI-MS/MS analysis.

Peak No.	GT No.	Adduct type	Identification	RT (min)	Detected mass (*m/z*)	Calculated mass (*m/z*)	Molecular formula	Characteristic fragmentation ions (*m/z*)
1		$[M+COOH]^-$	Sucrose	1.16	387.11438	387.11441	$C_{12}H_{22}O_{11}$	341.11($C_{12}H_{21}O_{11}$)
2		$[M+H]^+$	Quinic acid	1.19	191.05588	191.05611	$C_7H_{12}O_6$	109.03($C_6H_5O_2$)
3		$[M+H]^+$	Chlorogenic acid	6.96	355.10202	355.10236	$C_{16}H_{18}O_9$	163.04($C_9H_7O_3$)<br>145.03($C_9H_5O_2$)
4		$[M+H]^+$	Cryptochlorogenic acid	7.56	355.10226	355.10236	$C_{16}H_{18}O_9$	163.04($C_9H_7O_3$)
5		$[M+H]^+$	Isochlorogenic acid	8.96	355.10248	355.10236	$C_{16}H_{18}O_9$	163.04($C_9H_7O_3$)
6		$[M+H]^+$	Epicatechin	11.88	291.08618	291.08631	$C_{15}H_{14}O_6$	139.04($C_7H_7O_3$)<br>123.04($C_7H_7O_2$)
7		$[M+H]^+$	cis-5-Cafffeoylquinic acid	12.08	353.08774	353.08780	$C_{16}H_{18}O_9$	191.06($C_7H_{11}O_6$)<br>127.04($C_6H_7O_3$)
8		$[M+H]^+$	Gallocatechol C-glucoside	15.50	469.13144	469.13405	$C_{21}H_{24}O_{12}$	317.08($C_{13}H_{17}O_9$)
9		$[M+H]^+$	Rhynchophylloside A	15.64	563.22314	563.22353	$C_{27}H_{34}N_2O_{11}$	401.17($C_{21}H_{25}N_2O_6$)
10		$[M+H]^+$	Scopoletin	16.06	193.04961	193.04953	$C_{10}H_8O_4$	178.03($C_9H_6O_4$)<br>165.05($C_9H_9O_3$)

								150.03($C_8H_6O_3$)<br>133.03($C_8H_5O_2$)
11		$[M+H]^+$	22-O-Demethyl-22-O-β-glucopyranosyl isorhyn-chophylline	16.98	533.21289	533.24935	$C_{27}H_{36}N_2O_9$	371.20($C_{21}H_{27}N_2O_4$)<br>353.19($C_{21}H_{25}N_2O_3$)<br>269.16($C_{17}H_{21}N_2O$)<br>212.13($C_{11}H_{18}NO_3$)<br>160.08($C_{10}H_{10}NO$)
12		$[M+H]^+$	Corynoxinic acid	17.86	371.19666	371.19653	$C_{21}H_{26}N_2O_4$	353.19($C_{21}H_{25}N_2O_3$)<br>267.15($C_{17}H_{19}N_2O$)
13		$[M+H]^+$	22-O-Demethyl-22-O-β-glucopyranosyl iso-corynoxeine	18.11	531.23395	531.23371	$C_{27}H_{34}N_2O_9$	369.18($C_{21}H_{25}N_2O_4$)<br>160.08($C_{10}H_{10}NO$)<br>108.08($C_7H_{10}N$)
14		$[M+H]^+$	Rutin	19.36	611.16010	611.16066	$C_{27}H_{30}O_{16}$	303.05($C_{15}H_{11}O_7$)
15		$[M+H]^+$	Hyperoside	19.87	465.10248	465.10275	$C_{21}H_{20}O_{12}$	303.05($C_{15}H_{11}O_7$)
16		$[M+H]^+$	22-O-Demethyl-22-O-β-glucopyranosyl rhyncho-phylline	20.02	533.23859	533.24935	$C_{27}H_{36}N_2O_9$	371.20($C_{21}H_{27}N_2O_4$)<br>353.19($C21H_{25}N_2O_3$)<br>269.16($C_{17}H_{21}N_2O$)<br>212.13($C_{11}H_{18}NO_3$)<br>160.08($C_{10}H_{10}NO$)
17		$[M-H]^-$	Isomer of rutin	20.14	609.14587	609.14610	$C_{27}H_{30}O_{16}$	300.03($C_{15}H_8O_7$)<br>271.02($C_{14}H_7O_6$)<br>255.03($C_{14}H_7O_5$)
18		$[M+H]^+$	Vincosamide glucopyra-noside	20.56	839.30713	839.30805	$C_{38}H_{50}N_2O_{19}$	677.25($C_{29}H_{43}NO_{17}$)<br>515.20($C_{26}H_{31}N_2O_9$)<br>353.15($C_{20}H_{21}N_2O_4$)<br>283.11($C_{16}H_{15}N_2O_3$)
19		$[M+H]^+$	9-Hydroxy isocorynox-eine	21.07	399.22763	399.19144	$C_{22}H_{26}N_2O_5$	283.14($C17H_{19}N_2O_2$)<br>176.07($C10H_{10}NO_2$)<br>148.08($C_9H_{10}NO$)<br>108.08($C_7H_{10}N$)
20	GT20	$[M+H]^+$	Uncarine C	21.71	369.18036	369.18088	$C_{21}H_{24}N_2O_4$	267.15($C_{17}H_{19}N_2O$)
21		$[M+H]^+$	Hydrated cadambine	21.77	565.23865	565.23919	$C_{27}H_{36}N_2O_{11}$	548.21($C_{27}H_{34}NO_{11}$)<br>386.16($C21H_{24}NO_6$)<br>354.13($C_{17}H_{22}O_8$)
49		$[M+H]^+$	Cadambine	21.97	545.21283	545.21297	$C_{27}H_{32}N_2O_{10}$	383.16($C21H_{23}N_2O_5$)<br>351.12($C20H_{18}N_2O_4$)<br>263.11($C16H_{10}N_2O_2$)<br>227.12($C14H_{15}N_2O$)
22		$[M+H]^+$	Isomer of 11-hydroxy-2-O-D-glucopyranosyl vin-coside lactam	22.12	677.25409	677.25523	$C_{32}H_{40}N_2O_{14}$	515.20($C_{23}H_{33}NO_{12}$)<br>353.15($C_{20}H_{21}N_2O_4$)
23		$[M+H]^+$	Quercetin 3-rhamnoside	22.57	449.10770	449.10784	$C_{21}H_{20}O_{11}$	303.05($C_{15}H_{11}O_7$)
24		$[M+H]^+$	3α-Dihydrocadambine	23.30	547.22870	547.22862	$C_{27}H_{34}N_2O_{10}$	385.18($C_{21}H_{25}N_2O_5$)<br>367.16($C_{21}H_{23}N_2O_4$)<br>335.14($C_{20}H_{18}N_2O_3$)<br>317.13($C_{20}H_{17}N_2O_2$)
26		$[M+H]^+$	3β-Dihydrocadambine	24.05	547.22882	547.22862	$C_{27}H_{34}N_2O_{10}$	385.18($C_{21}H_{25}N_2O_5$)<br>367.16($C_{21}H_{23}N_2O_4$)<br>353.15($C_{20}H_{21}N_2O_4$)<br>335.14($C_{20}H_{18}N_2O_3$)
25	GT9	$[M+H]^+$	Corynoxine B	24.05	385.20132	385.21218	$C_{22}H_{28}N_2O_4$	353.19($C_{21}H_{25}N_2O_3$)<br>269.16($C_{17}H_{21}N_2O$)<br>226.14($C_{12}H_{20}NO_3$)<br>160.08($C_{10}H_{10}NO$)
27		$[M+H]^+$	vincoside lactam	24.14	499.20728	499.20749	$C_{26}H_{30}N_2O_8$	337.15($C_{20}H_{21}N_2O_3$)<br>319.14($C_{20}H_{19}N_2O_2$)<br>267.11($C_{16}H_{15}N_2O_2$)<br>171.09($C_{11}H_{11}N_2$)<br>144.08($C_{10}H_{10}N$)

28	GT37	$[M+H]^+$	vincoside lactam_qt	25.19	353.18591	353.18597	$C_{21}H_{24}N_2O_3$	353.19($C_{21}H_{25}N_2O_3$)<br>249.14($C_{17}H_{17}N_2$)<br>144.08($C_{10}H_{10}N$)
29	GT35	$[M+H]^+$	(1'R,3S,4a'S,5a'S,10a'R)-1'-methyl-2-oxo-1',4a',5',5a',7',8',10',10a'-octahydrospiro[indoline-3,6'-pyrano[3,4-f]indolizine]-4'-carboxylic acid	25.66	355.20111	355.16523	$C_{20}H_{22}N_2O_4$	338.18($C_{20}H_{22}N_2O_3$)
30		$[M+H]^+$	11-hydroxy-2-O-D-glucopyranosyl vincoside lactam	26.39	677.25452	677.25523	$C_{32}H_{40}N_2O_{14}$	515.20($C_{23}H_{33}NO_{12}$)<br>353.15($C_{20}H_{21}N_2O_4$)
31	GT19	$[M+H]^+$	Isorhynchophyllic acid	26.58	371.19629	371.19653	$C_{21}H_{26}N_2O_4$	353.14($C_{20}H_{21}N_2O_4$)<br>187.08($C_{12}H_{11}O_2$)<br>160.07($C_{10}H_{10}NO$)<br>142.06($C_{10}H_8N$)
32	GT16	$[M+H]^+$	Isocorynoxeine	26.69	383.19601	383.19653	$C_{22}H_{26}N_2O_4$	351.17($C_{21}H_{23}N_2O_3$)<br>319.14($C_{20}H_{19}N_2O_2$)<br>267.15($C_{17}H_{19}N_2O$)<br>201.10($C_{12}H_{13}N_2O$)<br>160.08($C_{10}H_{10}NO$)
33		$[M+H]^+$	Isocorynoxine B	26.69	385.29199	385.21218	$C_{22}H_{28}N_2O_4$	353.19($C_{21}H_{25}N_2O_3$)<br>321.16($C_{20}H_{21}N_2O_2$)<br>160.08($C_{10}H_{10}NO$)
34		$[M+H]^+$	3-Epistrictosidine	26.87	531.23309	531.23371	$C_{27}H_{34}N_2O_9$	514.21($C_{27}H_{32}NO_9$)<br>352.15($C_{21}H_{22}NO_4$)<br>334.14($C_{21}H_{20}NO_3$)
35		$[M-H]^-$	3,5-Dicaffeoylquinic acid	26.95	515.11938	515.11950	$C_{25}H_{24}O_{12}$	353.09($C_{16}H_{17}O_9$)<br>173.05($C_7H_9O_5$)
36		$[M+H]^+$	9-Hydroxy corynoxeine	27.07	399.19083	399.19144	$C_{22}H_{26}N_2O_5$	367.16($C_{21}H_{23}N_2O_4$)<br>176.07($C_{10}H_{10}NO_2$)<br>158.06($C_{10}H_8NO$)<br>108.08($C_7H_{10}N$)
37		$[M+H]^+$	Strictosidine	27.46	531.23389	531.23371	$C_{27}H_{34}N_2O_9$	514.21($C_{27}H_{32}NO_9$)<br>352.15($C_{21}H_{22}NO_4$)<br>334.14($C_{21}H_{20}NO_3$)
38	GT29	$[M+H]^+$	geissoschizinc acid	28.00	353.18619	353.18597	$C_{21}H_{24}N_2O_3$	291.15($C_{19}H_{19}N_2O$)
39	GT41	$[M+H]^+$	isocorynantheic acid	28.21	353.18573	353.18597	$C_{21}H_{24}N_2O_3$	291.15($C_{19}H_{19}N_2O$)
40	GT24	$[M+H]^+$	Isorhynchophylline	28.24	385.21246	385.21218	$C_{22}H_{28}N_2O_4$	353.19($C_{21}H_{25}N_2O_3$)<br>321.16($C_{20}H_{21}N_2O_2$)<br>241.13($C_{15}H_{17}N_2O$)<br>226.14($C_{12}H_{20}NO_3$)<br>160.08($C_{10}H_{10}NO$)
41	GT7	$[M+H]^+$	Corynoxeine	28.51	383.19690	383.19653	$C_{22}H_{26}N_2O_4$	351.17($C_{21}H_{23}N_2O_3$)<br>319.14($C_{20}H_{19}$N2O2)<br>267.15($C_{17}H_{19}N_2O$)<br>201.10($C_{12}H_{13}N_2O$)<br>160.08($C_{10}H_{10}NO$)
42		$[M+H]^+$	Isocorynoxeine N-oxide	28.94	399.19135	399.19144	$C_{22}H_{26}N_2O_5$	383.20($C_{22}H_{27}N_2O_4$)<br>206.12($C_{12}H_{16}NO_2$)<br>160.08($C_{10}H_{10}NO$)<br>108.08($C_7H_{10}N$)
43	GT40	$[M+H]^+$	Yohimbin	28.95	355.20178	355.20162	$C_{21}H_{26}N_2O_3$	337.19($C_{21}H_{25}N_2O_2$)<br>224.13($C_{12}H_{17}NO_3$)<br>144.08($C_{10}H_{10}N$)
44	GT42	$[M+H]^+$	β-Yohimbin	29.84	355.20154	355.20162	$C_{21}H_{26}N_2O_3$	338.17($C_{21}H_{24}N_2O_2$)<br>224.13($C_{12}H_{17}NO_3$)<br>144.08($C_{10}H_{10}N$)
45		$[M+H]^+$	NEOCHLOROGENIC ACID	3.84	355.10211	355.10236	$C_{16}H_{18}O_9$	163.04($C_9H_7O_3$)<br>145.03($C_9H_5O_2$)

46		$[M+H]^+$	Ajmalicine	30.66	353.18573	353.18597	$C_{21}H_{24}N_2O_3$	321.16($C_{20}H_{21}N_2O_2$)<br>222.11($C_{12}H_{15}NO_3$)<br>210.11($C_{11}H_{16}NO_3$)<br>144.08($C_{10}H_{10}N$)
47	GT8	$[M+H]^+$	Corynoxine	30.79	385.21179	385.21218	$C_{22}H_{28}N_2O_4$	241.13($C_{15}H_{17}N_2O$)<br>160.08($C_{10}H_{10}NO$)
48	GT25	$[M+H]^+$	Isomitraphylline	31.21	369.21722	369.18088	$C_{21}H_{24}N_2O_4$	336.18($C_{21}H_{24}N_2O_2$)<br>158.06($C_{10}H_8NO$)
50	GT33	$[M+H]^+$	Mitraphyllic acid	32.43	355.20114	355.16523	$C_{20}H_{22}N_2O_4$	144.08($C_{10}H_{10}N$)<br>108.08($C_7H_{10}N$)
51		$[M+H]^+$	Geissoschizine	32.58	353.18594	353.18597	$C_{21}H_{24}N_2O_3$	304.13($C_{20}H_{18}O_2N$)<br>222.11($C_{12}H_{15}NO_3$)<br>210.11($C_{11}H_{16}NO_3$)<br>144.08($C_{10}H_{10}N$)
52		$[M+H]^+$	3β-Isodihydrocadam-bine	33.64	547.22888	547.22862	$C_{27}H_{34}N_2O_{10}$	385.18($C_{21}H_{25}N_2O_5$)<br>367.16($C_{21}H_{23}N_2O_4$)<br>349.15($C_{21}H_{20}N_2O_3$)
53	GT23	$[M+H]^+$	Vallesiachotamine	33.72	351.17032	351.17032	$C_{21}H_{22}N_2O_3$	335.14($C_{20}H_{18}N_2O_3$)<br>319.14($C_{20}H_{19}N_2O_2$)<br>249.14($C_{17}H_{17}N_2$)
55	GT5	$[M+H]^+$	Corynantheine	33.92	367.20169	367.20162	$C_{22}H_{26}N_2O_3$	251.15($C_{17}H_{19}N_2$)<br>236.12($C_{13}H_{17}NO_3$)<br>224.13($C_{12}H_{18}NO_3$)<br>192.10($C_{11}H_{14}NO_2$)
54	GT31	$[M+H]^+$	methyl (E)-2-[(2S,3Z,12bS)-3-ethyli-dene-2,4,6,7,12,12b-hex-ahydro-1H-indolo[3,2-h]quinolizin-2-yl]-3-methoxyprop-2-enoate	33.92	367.20126	367.20162	$C_{22}H_{26}N_2O_3$	144.08($C_{10}H_{10}N$)
56	GT26	$[M+H]^+$	Mitraphylline	34.08	369.21683	369.18088	$C_{21}H_{24}N_2O_4$	336.18($C_{21}H_{24}N_2O_2$)<br>158.06($C_{10}H_8NO$)
57	GT4	$[M+H]^+$	Angustoline	34.54	332.13904	332.13935	$C_{20}H_{17}N_3O_2$	316.11($C_{19}H_{14}N_3O_2$)
58	GT2	$[M+H]^+$	akuammigine	35.36	353.18570	353.18597	$C_{21}H_{24}N_2O_3$	335.22($C_{21}H_{23}N2O2$)<br>222.67($C_{12}H_{16}NO_3$)<br>172.31($C_{10}H_{24}N_2$)
59	GT14	$[M+H]^+$	Hirsuteine	35.39	367.20139	367.20162	$C_{22}H_{26}N_2O_3$	251.15($C_{17}H_{19}N_2$)<br>224.13($C_{12}H_{18}NO_3$)
60	GT15	$[M+H]^+$	Hirsutine	35.89	369.21713	369.21727	$C_{22}H_{28}N_2O_3$	336.18($C_{21}H_{24}N_2O_2$)<br>144.08($C_{10}H_{10}O$)
61	GT30	$[M+H]^+$	Rhynchophylline A	36.78	353.18594	353.18597	$C_{21}H_{24}N_2O_3$	210.11($C_{11}H_{16}NO_3$)<br>144.08($C_{10}H_{10}N$)<br>108.08($C_7H_{10}N$)
62		$[M+H]^+$	Isomer of vincoside lac-tam	36.95	499.20718	499.20749	$C_{26}H_{30}N_2O_8$	337.15($C_{20}H_{21}N_2O_3$)<br>319.14($C_{20}H_{19}N_2O_2$)<br>267.11($C_{16}H_{15}N_2O_2$)<br>171.09($C_{11}H_{11}N_2$)<br>144.08($C_{10}H_{10}N$)
63	GT27	$[M+H]^+$	Speciophylline/Un-carine D	38.42	369.21725	369.18088	$C_{21}H_{24}N_2O_4$	160.08($C_{10}H_{10}NO$)
64		$[M+H]^+$	Sitsirikine	38.55	355.20190	355.20162	$C_{21}H_{26}N_2O_3$	224.14($C_{12}H_{17}NO_3$)<br>212.13($C_{11}H_{18}NO_3$)<br>144.08($C_{10}H_{10}N$)
65	GT6	$[M+H]^+$	Corynanthine	38.61	355.20132	355.20162	$C_{21}H_{26}N_2O_3$	144.08($C_{10}H_{10}N$)
66		$[M+H]^+$	Pubescin	39.76	383.19623	383.19653	$C_{22}H_{26}N_2O_4$	223.12($C_{15}H_{15}N_2$)<br>184.10($C_{12}H_{12}N_2$)
67	GT11	$[M+H]^+$	Geissoschizine methyl ether	40.08	367.20541	367.20162	$C_{22}H_{26}N_2O_3$	249.14($C_{17}H_{17}N_2$)
68		$[M-H]^-$	Quinovic acid diglyco-side	40.35	809.30121	809.43289	$C_{42}H_{66}O_{15}$	603.39($C_{28}H_{59}O_{13}$)

69	GT21	$[M+H]^+$	Rhynchophylline	40.98	385.21219	385.21218	$C_{22}H_{28}N_2O_4$	353.19($C_{21}H_{25}N_2O_3$)<br>321.16($C_{20}H_{21}N_2O_2$)<br>226.14($C_{12}H_{20}NO_3$)<br>160.08($C_{10}H_{10}NO$)
70		$[M+H]^+$	Strictosamide	41.18	499.20691	499.20749	$C_{26}H_{30}N_2O_8$	337.15($C_{20}H_{21}N_2O_3$)<br>319.14($C_{20}H_{19}N_2O_2$)<br>267.11($C_{16}H_{15}N_2O_2$)<br>171.09($C_{11}H_{11}N_2$)<br>144.08($C_{10}H_{10}N$)
71	GT10	$[M+H]^+$	Dihydrocorynantheine	41.31	369.21710	369.21727	$C_{22}H_{28}N_2O_3$	251.15($C_{17}H_{19}N_2$)<br>238.15($C_{13}H_{19}NO_3$)<br>226.14($C_{12}H_{20}NO_3$)
72	GT18	$[M+H]^+$	Isopteropodine	41.62	369.21667	369.18088	$C_{21}H_{24}N_2O_4$	352.19($C_{21}H_{23}N_2O_3$)
73	GT28	$[M+H]^+$	Uncarine F	41.67	369.18066	369.18088	$C_{21}H_{24}N_2O_4$	226.14($C_{12}H_{20}NO_3$)
74		$[M+H]^+$	Mitragynine	42.09	399.18942	399.22783	$C_{23}H_{30}N_2O_4$	367.16($C_{21}H_{23}N_2O_4$)<br>160.08($C_{10}H_{10}NO$)
75	GT22	$[M+H]^+$	Tetrahydroalstonine	42.77	353.18619	353.18597	$C_{21}H_{24}N_2O_3$	335.22($C_{21}H_{23}N_2O_2$)<br>222.67($C_{12}H_{16}NO_3$)<br>172.31($C_{10}H_{24}N_2$)
76		$[M+H]^+$	Rhynchophylline N-oxide	43.57	401.20663	401.20709	$C_{22}H_{28}N_2O_5$	383.20($C_{22}H_{27}N_2O_4$)<br>369.18($C_{21}H_{25}N_2O_4$)<br>351.17($C_{21}H_{23}N_2O_3$)
77	GT13	$[M+H]^+$	Harman	47.23	183.13766	183.09167	$C_{12}H_{10}N_2$	105.03($C_6H_5N_2$)
78	GT36	$[M+H]^+$	(2S,12bR)-methyl 2-((E)-1-oxobut-2-en-2-yl)-1,2,6,7,12,12b-hexahydroindolo[2,3-a]quinolizine-3-carboxylate	47.81	351.17017	351.17032	$C_{21}H_{22}N_2O_3$	319.1438($C_{20}H_{19}N_2O_2$)
79	GT3	$[M+H]^+$	Angustidine	48.45	302.30521	302.12879	$C_{19}H_{15}N_3O$	70.07(C_4H_8N)
80		$[M+H]^+$	Quinovic acid	49.22	487.21490	487.34180	$C_{30}H_{46}O_5$	451.32($C_{30}H_{43}O_3$)<br>423.33($C_{29}H_{43}O_2$)

3. Supplementary Figure

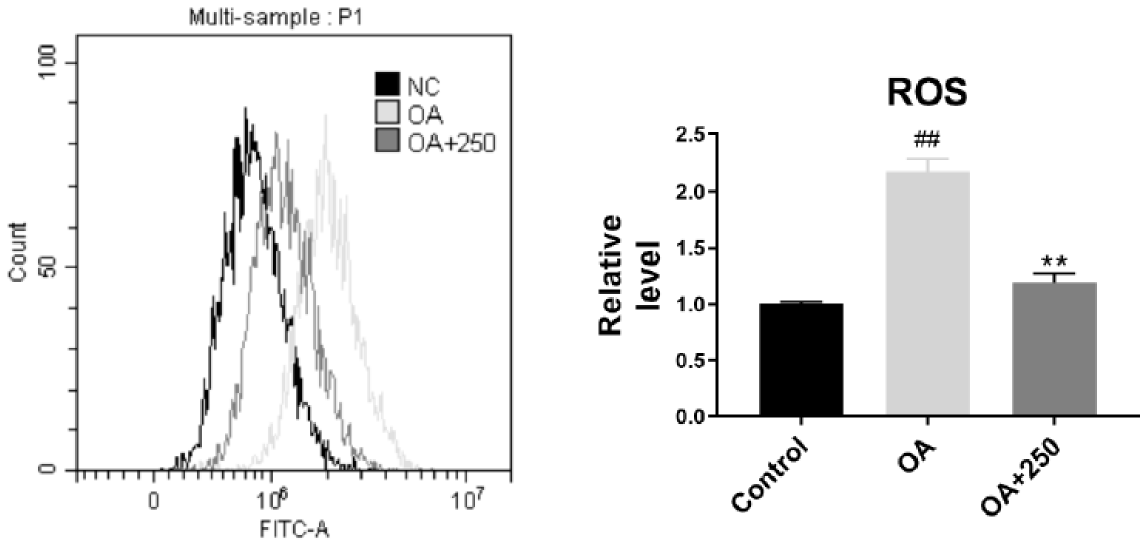

Figure S1. The effect of GT extract (250 μg/mL) for relative ROS level on OA-induced SH-SY5Y cells. ## $p < 0.01$ compared with control group, ** $p < 0.01$, compared with model group.
